# Microhomologies are prevalent at Cas9-induced larger deletions

**DOI:** 10.1093/nar/gkz459

**Published:** 2019-05-25

**Authors:** Dominic D G Owens, Adam Caulder, Vincent Frontera, Joe R Harman, Alasdair J Allan, Akin Bucakci, Lucas Greder, Gemma F Codner, Philip Hublitz, Peter J McHugh, Lydia Teboul, Marella F T R de Bruijn

**Affiliations:** 1 MRC Molecular Hematology Unit, MRC Weatherall Institute of Molecular Medicine, Radcliffe Department of Medicine, University of Oxford, Oxford OX3 9DS, UK; 2 The Mary Lyon Centre, MRC Harwell Institute, Didcot, Oxon OX11 0RD, UK; 3 WIMM Genome Engineering Facility, MRC Weatherall Institute of Molecular Medicine, Radcliffe Department of Medicine, University of Oxford, Oxford OX3 9DS, UK; 4 Department of Oncology, MRC Weatherall Institute of Molecular Medicine, University of Oxford, Oxford OX3 9DS, UK

## Abstract

The CRISPR system is widely used in genome editing for biomedical research. Here, using either dual paired Cas9^D10A^ nickases or paired Cas9 nuclease we characterize unintended larger deletions at on-target sites that frequently evade common genotyping practices. We found that unintended larger deletions are prevalent at multiple distinct loci on different chromosomes, in cultured cells and mouse embryos alike. We observed a high frequency of microhomologies at larger deletion breakpoint junctions, suggesting the involvement of microhomology-mediated end joining in their generation. In populations of edited cells, the distribution of larger deletion sizes is dependent on proximity to sgRNAs and cannot be predicted by microhomology sequences alone.

## INTRODUCTION

CRISPR/Cas9-based genome engineering approaches are widely used to generate deletions or insertions at genomic regions of interest for biomedical research purposes. Cas9 nuclease (derived from *Streptococcus pyogenes*) generates double strand DNA breaks (DSBs) when targeted to a locus by a single-guide RNA (sgRNA) that facilitates base pairing with the DNA template, recruiting the nuclease on-target (1). Although targeting is primarily specific, off-target sites, which differ by one or more bases, may also be recognised and cut (1). To reduce off-target editing, modified approaches have been developed including high-fidelity Cas9 (hfCas9) (2), and Cas9 nickase (3). Two different Cas9 nickase enzymes were engineered, Cas9^D10A^ and Cas9^H840A^, which each harbour inactivating mutations in one of the two Cas9 nuclease domains and generate single stranded DNA (ssDNA) nicks upon recruitment to the DNA (3). To generate a DSB at the locus of interest, Cas9 nickase is targeted using two sgRNA molecules (paired sgRNAs) that yield two ssDNA nicks in close proximity on opposite strands, resulting in staggered DSB formation (3). At off-target sites, the likelihood of two sgRNAs binding is small, thus resulting in ssDNA nicks that are repaired with high efficiency by the mismatch repair pathway (4). Because of its reduced probability of off-target mutations, Cas9 nickase-mediated genome editing has been suggested to be favourable for gene therapy approaches (5).

For the deletion of a specific genomic region using Cas9 nuclease or Cas9 nickase, two (paired) or four (dual paired) sgRNAs are typically used, respectively, to generate DSBs flanking a target region. DSBs can be repaired through several endogenous repair pathways including non-homologous-end-joining (NHEJ), homologous recombination (HR), microhomology-mediated end joining (MMEJ) or single strand annealing (SSA) (1,6,7). In some alleles, DNA repair will result in loss of the intervening DNA between flanking DSBs. Such deletions are commonly detected by short-range (S-R) polymerase chain reaction (PCR) and Sanger sequencing (8,9). In this approach, flanking primers adjacent to the target region (typically ≤200 bp away from sgRNA cut sites) are used to amplify genomic DNA (gDNA) of targeted cells or tissues (10,11). DSB repair can also result in additional insertions or deletions (indels). Indels generated by Cas9 are typically small in size (<50 bp) (12–16), but larger deletions (LDs) have also been reported. For example, larger than expected indels from one sgRNA spanned from ∼300 to 9.5 kb in HEK-293T or mouse embryonic stem cells (mESCs) (12,17). Paired sgRNAs also induced LDs in MEL cells (18). When genome editing *in vivo*, LDs ranging from hundreds of bp to several kb were seen in up to 45% of mouse or rat embryos edited with single or paired sgRNAs (8,19–27). The majority of LDs previously identified were induced by Cas9 nuclease but have also been observed at a single locus targeted with dual Cas9 nickase complexes in mouse embryos (21). Such unanticipated LDs may be difficult to detect using S-R PCR screening methods, as primer binding sites may be lost, resulting in a failure to amplify these alleles (17,24). It remains unclear how prevalent or widespread LDs are at different loci, or when using different CRISPR/Cas9 genome editing modalities such as Cas9 nickase. Moreover, the DNA repair mechanisms at play for LD generation remain unclear.

Here, we performed genome editing using dual paired Cas9^D10A^ nickase, paired Cas9 nuclease, and single Cas9 nuclease complexes in mESCs and a haematopoietic progenitor cell line *in vitro*, and in mouse embryos *in vivo*. Sixteen separate regions ranging from 100 bp to 1.5 kb corresponding to both coding and non-coding regions were targeted at nine different genomic loci on seven chromosomes. Using PCR and droplet digital PCR, we identified LDs of up to 7 kb from paired sgRNA target sites and sequenced the breakpoint junctions. Significant microhomologies consistent with MMEJ were detected at almost all LD breakpoint junctions. Our findings show that LDs are not repaired at the most proximal microhomologies. Instead, using computational approaches we show that the distribution of LDs in a cell population can be modelled based on proximity to sgRNA cut sites.

## MATERIALS AND METHODS

### Cell culture

The 416B myeloid progenitor cell line (28) was grown in Fischers medium (Gibco) supplemented with 20% horse serum (Gibco), 2 mM l-glutamine. Cells were maintained at 37°C and 5% CO_2_, at densities of between 2 × 10^5^ and 8 × 10^5^ cells/ml. E14-TG2a (29) and E14-TG2a-RV mESCs (stably transfected with a Venus reporter at the 3′ end of *Runx1* and a hsp68-mCherry-Runx1+23 enhancer-reporter transgene in the *Col1a1* locus; L Greder, unpublished data) were cultured in GMEM medium (Gibco) supplemented with 10% FCS (Gibco), 2% Leukemia Inhibitory Factor (LIF) conditioned medium, 2 mM l-glutamine (Gibco) and 100 μM β-mercaptoethanol (Sigma). Cells were passaged every 2–3 days.

### Genome editing in cultured cells

Dual-Cas9^D10A^ nickase and Cas9 nuclease-based knock-out strategies were designed using the Zhang lab online tool (crispr.mit.edu). Single guide RNAs (Supplementary Table S1) were ordered as oligonucleotides (IDT) and cloned into the BbsI site of pX335-Neo (30) or pX459 (Addgene plasmid #62988, (31)). Gibson assembly was used to create tandem constructs that contained one, two or four sgRNAs in one plasmid. DH10β chemically competent *Escherichia coli* (Invitrogen) were transformed with plasmid DNA according to the manufacturer's instructions. Correct sgRNA inserts were confirmed by Sanger sequencing. Purified plasmids (Qiagen plus Midi prep kit (Qiagen)) were transfected into mESCs using lipofectamine 2000 (Invitrogen, 5 μg/well of a 6-well plate). 1 × 10^7^ 416B cells were electroporated with 10 μg of a modified pX335 plasmid co-expressing eGFP with a Bio-Rad gene pulser (Bio-Rad, 40mm cuvette, 220 mV, 960 μFD). Cells were either FACS sorted based on GFP expression or selected using 1 μg/ml puromycin or 175 μg/ml G418 (Gibco). Colony picking of mESCs was performed as previously described (32). DNA was purified from bulk populations of selected or sorted mESCs or sorted 416B cells using a Qiagen DNeasy blood and tissue kit (Qiagen).

### Mice

All animals were housed and maintained in the Mary Lyon Centre, MRC Harwell Institute under specific-pathogen-free (SPF) conditions, in individually ventilated cages adhering to environmental conditions as outlined in the Home Office Code of Practice. Mice were euthanized by Home Office Schedule 1 methods.

### Reagents for microinjection, delivery to embryos and germline transmission

Guide sequence selection was carried out using two online tools: CRISPOR (33) and Wellcome Trust Sanger Institute (WTSI) Genome Editing (WGE) (34). sgRNA sequences were selected with as few predicted off-target events as possible, particularly on the same chromosome as the intended modification (Sequences shown in Supplementary Table S1). Two sgRNAs for each side of the critical regions to be deleted were synthesized and co-injected with Cas9 mRNA as previously described (27). Injected embryos were re-implanted in CD-1 pseudopregnant females. Host females were allowed to litter and rear G_0_s. G_0_ animals where the presence of a desired allele was detected by PCR were mated to wild-type animals to obtain G_1_ animals for germline transmission of the allele of interest and definitive validation of its integrity.

### PCR analysis of cultured cells

PCR products were amplified from gDNA isolated from clones or pools of cells as indicated. PCR was performed using a HotStar Taq master mix kit (Qiagen), with 100 ng gDNA and primers at 200 nM (Primers listed in Supplementary Table S2). PCR products were analysed by agarose gel electrophoresis and a 1 kb plus ladder (Thermo Fischer). PCR products were gel extracted using a Zymoclean gel extraction kit (Zymo Research). TA cloning was performed according to the manufacturer's instructions (Invitrogen). Plasmids were isolated using a Qiagen Spin mini-prep kit (Qiagen) followed by Sanger sequencing (Source Bioscience, Oxford, UK).

### PCR analysis of mice

Genomic DNA was extracted from ear clip biopsies using the DNA Extract All Reagents Kit (Applied Biosystems) according to the manufacturer's instructions. Genotyping primers for SR-PCR assays were chosen to be at least 200 bp away from the sequences targeted by sgRNA, depending on available sequences for design. PCR assays were optimised and performed as previously described (27). The PCR products were purified employing a QIAquick Gel Extraction Kit (Qiagen) and sent for Sanger sequencing.

### Alignment of larger deletion alleles

All deletions were aligned to the mm9 reference genome using UCSC BLAT and visualised using the UCSC genome browser (35,36). Fine mapping was performed by subsequent local alignment using MUSCLE (37). Repetitive elements were mapped using UCSC RepeatMasker (38).

### Droplet digital PCR

Droplet Digital PCR was used to determine copy number variation in genome-edited mice and to quantify deletions in edited mESCs. Experiments were performed as duplex reactions, where the sequence employed as a donor was amplified using a fluorescein amidite (FAM)-labelled assay selected from a Universal Probe Library (UPL) set (Human, sourced from Roche, Basel, SZ). Suitable probes and primers were identified using the ProbeFinder software at the Roche assay design centre (accessible from www.universalprobelibrary.com, Supplementary Table S2). In cases where a UPL set was not available, custom assays were ordered from LGC Biosearch Technologies (Novato, USA). UPL or custom assays were used in parallel with a VIC-labelled reference gene assay (Dot1l, sourced from ThermoFisher) set at two copies (CNV2) on the Bio-Rad QX200 ddPCR System (Bio-Rad) as per Codner *et al.* (27). Reaction mixes (22 μl) contained 2 μl crude DNA lysate or 50 ng of phenol/chloroform purified genomic DNA, 1× ddPCR Supermix for probes (Bio-Rad), 225 nM of each primer (two primers per assay) and 50 nM of each probe (one VIC-labelled probe for the reference gene assay and one FAM-labelled for the target genomic region assays). For deletion quantification in pools of cells, 100 ng purified DNA was used. These reaction mixes were loaded either into DG8 cartridges together with 70 μl droplet oil per sample and the droplets generated using the QX100 Droplet Generator or loaded in plate format into the Bio-Rad QX200 AutoDG and the droplets generated as per the manufacturer's instructions. Post droplet generation, the oil/reagent emulsion was transferred to a 96-well semi-skirted plate (Eppendorf), and the samples were amplified on a Bio-Rad C1000 Touch thermocycler (95°C for 10 min, followed by 40 cycles of 94°C for 30 s and 58°C for 60 s, with a final elongation step of 98°C for 10 min, where all temperature ramping was set to 3°C/s). The plate containing the droplet amplicons was subsequently loaded into the QX200 Droplet Reader (Bio-Rad). Standard reagents and consumables supplied by Bio-Rad were used, including cartridges and gaskets, droplet generation oil and droplet reader oil. Copy numbers were assessed using the QuantaSoft Analysis Pro™ software using at least 10 000 accepted droplets per sample. The copy numbers (mice) or ratio compared to internal control (mESCs) were calculated by applying Poisson statistics to the fraction of end-point positive reactions, and the 95% confidence interval of this measurement is shown. When visualizing ddPCR quantification in pools of cells the mean and 95% confidence intervals are shown.

### Linear regression modelling of ddPCR data

Multiple linear regression was performed in R using the ‘lm’ and ‘predict’ functions. The model was fit using the formula ‘*y* ∼ log(*x*) + *a*’, where ‘*y*’ (frequency of deletion) was the response variable, ‘*x*’ (proximity to sgRNAs) was explanatory variable one and ‘a’ (sgRNA cutting efficiency) explanatory variable two. Data used to fit the model were empirically determined by ddPCR in eight different targeted cell populations and at two different loci. Frequency of deletion at a particular region was calculated by dividing the relative concentration determined by ddPCR in the transfected sample by the corresponding non-targeting control. Proximity to sgRNA binding sites was determined from the midpoints of the ddPCR amplicon and sgRNA. The sgRNA cutting efficiency in each sample was the highest frequency of deletion directly at a sgRNA target site as determined by ddPCR. For model estimates of deletion frequency, cutting efficiency was either set to the average cutting efficiency for all samples (when visualising general estimates) or a known cutting efficiency when estimating values in a subset of the experiments. Goodness of fit testing was performed by plotting a histogram of residuals (where residual = observed value - predicted value), and a *Q*–*Q* plot (quantile–quantile plot) to check that residuals are normally distributed (which is an assumption of the linear regression model). The distribution of estimated relative allele frequencies (1 – deletion frequency) was plotted across a 3 kb window up and downstream of a simulated sgRNA cut site.

### Microhomology scoring and quantification

Deletions were considered LDs if they spanned >200 bp from sgRNA target sites and ablated at least one S-R primer binding site. All of the larger deletions that could be resolved by PCR and Sanger sequencing were quantified (Supplementary Table S5). Deletions were considered to be of expected size if indels reached ≤25 bp beyond the protospacer adjacent motif (PAM) site of the nearest sgRNA binding site. LDs previously described in the literature (8,19–24) were considered if they spanned >80 bp away from predicted sgRNA cut sites. Microhomology scoring was performed using a custom R script and the package Biostrings. 10 bp in the 5′ and 3′ direction at up and downstream breakpoints was used to search for microhomologies. Identical bases were given a score of 1 and mismatches were given a score of 0. The highest scoring homology at each breakpoint site (either upstream or downstream) was considered. Bases were considered homologous if they were identical and directly abutted the deletion, as previously described (39). To simulate the background distribution of microhomologies in the genome, random genomic locations were selected using a custom R script and the package BSgenome. The length of the simulated LDs was set to the average length of LDs identified. Chance expectation of homology at any two locations for a *k*-mer of a given length was calculated as previously described (40) using the equation *P*(*x*) = (*x* + 1)(1/4)^*x*^(3/4)^2^. Alternative microhomologies were counted within deleted sequences (excluding sequences between paired sgRNAs) using R and Biostrings. Statistical analysis was performed in graph pad prism and R using χ^2^ test, Kruskal–Wallis with Dunn's multiple comparison test, or two-tailed Mann–Whitney test as indicated. All custom scripts are freely available on GitHub (https://github.com/d0minicO/mhscanR).

### Deep sequencing of short deletions in CRISPR/Cas9 edited cells

Locus specific next-generation sequencing primers (NGS, Supplementary table S2) were designed to amplify a 225 bp amplicon centred on each sgRNA cut site. Primers were modified to contain Illumina Truseq adapter sequences at the 5′ end. PCR was performed (25 cycles, Herculase II PCR kit (Agilent)) on a pool of genomic DNA harvested from cells 3–7 days post transfection with one sgRNA. Truseq indices (NEB E7335) were added to the PCR amplified fragments by using a further 6 cycles of PCR with Herculase II PCR kit. The size distribution of libraries was analysed using a D1000 Tapestation (Agilent) and library concentration was quantified using KAPA library quantification kit (Roche) both according to the manufacturer's instructions. Libraries were normalised to 4nM and pooled before sequencing using Illumina MiSeq v2 300 cycle paired end kit. Raw fastq files have been deposited in NCBI’s Gene Expression Omnibus (41) and are accessible through GEO Series accession number GSE130621 (https://www.ncbi.nlm.nih.gov/geo/query/acc.cgi?acc=GSE130621). Fastq files were trimmed using trim_galore. For visualization, trimmed fastq files were flashed (42) and mapped using bwa mem v 0.7.12. Sam files were converted to bam using samtools (43), converted to bigwig using deeptools bamCoverage (option -bs 1) (44), and visualised using UCSC genome browser. For analysis of individual alleles trimmed fastq files were analysed using CRISPResso v1.0.8 (45). A custom R script was used to quantify microhomologies in simple deletions (spanning a contiguous region and free of insertions or mutations within 10 bp of the deletion) using the same microhomology scoring criteria as at LDs. Reads containing insertions were quantified against the background of all modified reads. Graphing and χ^2^ tests were performed in R and graph pad prism.

### Analysis of GC content at microhomologies

GC content of microhomologies was analysed as previously described (46). Briefly, in the absence of GC bias, the GC content of a microhomology is assumed equal to the background GC content over the region containing the deletions. The background GC content in deep sequencing data was calculated over the region containing 93–95% of the reads. The observed GC base pair content of microhomologies was then compared to the expected probability using the χ^2^ test (Graph Pad Prism, or R).

## RESULTS

### Genome editing with Cas9^D10A^ nickase in mESCs causes larger than expected on-target deletions

**Figure 1.**
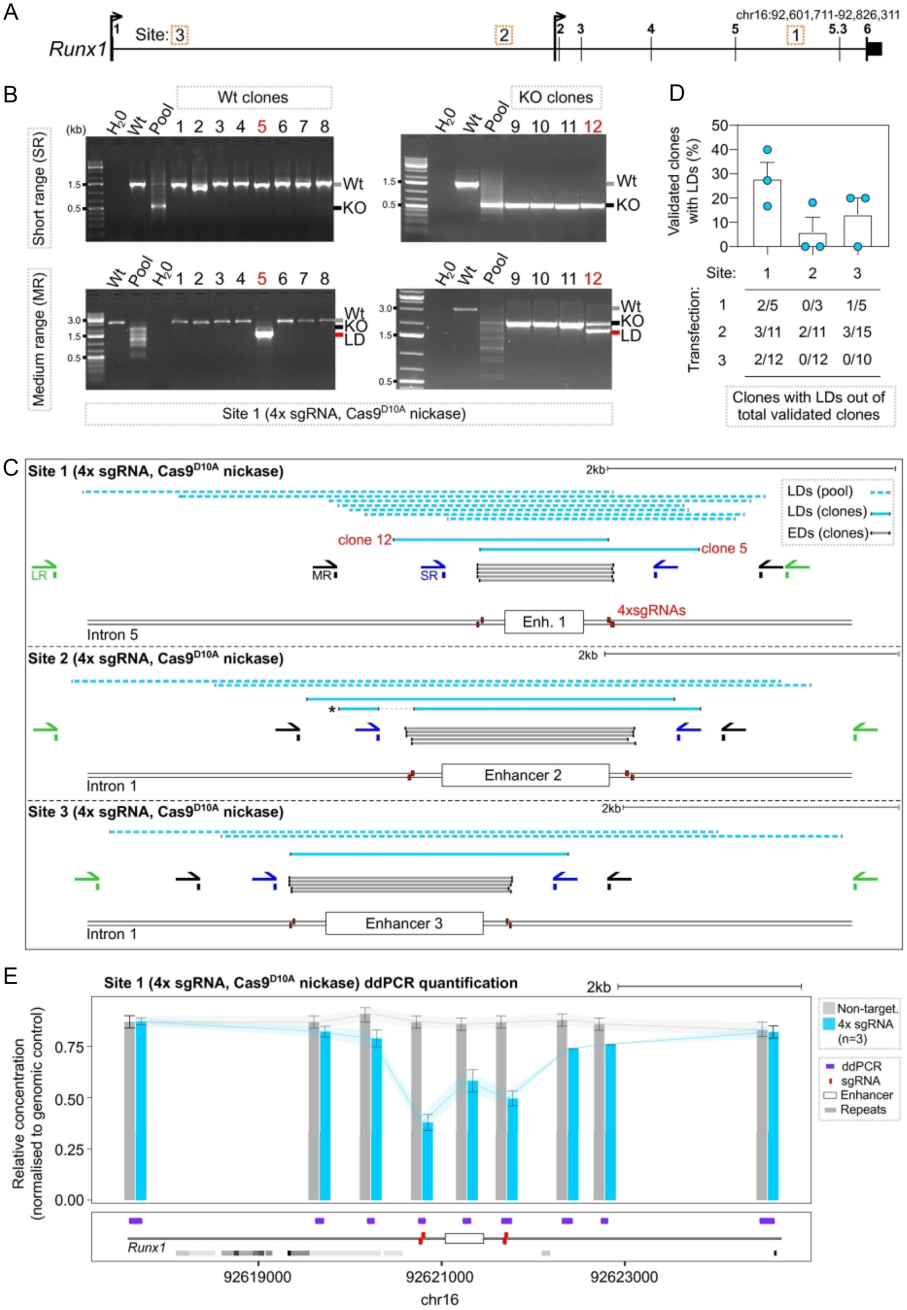
Characterisation of larger deletions at three sites targeted by CRISPR/Cas9^D10A^ nickase. (**A**) Locus map of the *Runx1* gene showing the positions of evolutionarily conserved cis-regulatory elements (Site 1–3, corresponding to Enhancer 1–3) that were targeted in E14-TG2a-RV mESCs using CRISPR/Cas9^D10A^ nickase. (**B**) Example gel images from one experiment targeting Site 1. Gel images show PCR amplification from gDNA of isolated wild type (wt) clones (left hand gels) and knock-out (KO) clones (right hand gels) with SR primers (top gels) and MR primers (bottom gels). Wt next to the gel image indicates the size of the wild type allele, KO indicates the size of alleles harbouring the expected deletion, and LD indicates the size of alleles in clones identified as harbouring LDs. (**C**) Schematic showing the positions of S-R PCR primers (SR, blue), M-R PCR primers (MR, black), L-R PCR primers (LR, green), sgRNAs (red boxes), and LDs isolated from clones (dark blue lines) and pools of cells (light blue dashed lines) at each of the three sites. The allele marked with a star contained a secondary deletion at Site 2 distal to the primary cut site that destroyed a primer binding site. (**D**) Quantification of clone frequencies with homozygous wild type or knock-out genotypes by S-R PCR (validated clones) that were later found to contain a LD on another allele by M-R PCR. Quantification of clone numbers for each transfection that were homozygous knock-out or wild type and contained a LD on the other allele (*n* = 3 independent transfections per site, each dot is one independent experiment). (**E**) ddPCR quantification of deletions across a 7 kb window centred on Enhancer 1. Each bar represents the mean relative concentration of the target DNA sequence (±95% confidence interval). mESCs were targeted with 4× sgRNA (blue bars, *n* = 3) and a non-targeting control (grey bar).

We used a dual paired sgRNA (4x sgRNA) CRISPR/Cas9^D10A^ nickase strategy in mESCs that had been stably transfected with fluorescent reporters (E14-TG2a-RV) to delete evolutionarily conserved *Runx1 cis*-regulatory elements (47,48) (Figure 1A). Intended deletions ranged from 1 to 1.5 kb. Individual clones were analysed for the desired genotype using S-R PCR (Figure 1B, C). Out of 445 clones analysed, an average of 35% and 20% of the total isolated clones for each of the three targeted sites appeared to be homozygous knock-out or wild type, respectively (Supplementary Figure S1A–D). Several clones with unique alleles harbouring deletions of expected size (EDs, spanning <25 bp from expected sgRNA cut sites) were mapped using Sanger sequencing of PCR products (Figure 1B, C, grey lines). Sanger sequencing often generated a single sequencing trace, indicative either of an *iso*-allelic HR event (both alleles carrying the same deletion) or loss of a primer binding site, leading to failure to amplify one of the alleles (allelic drop-outs) (Supplementary Figure S1E).

To investigate the genotypes of clones with possible allelic drop-outs we performed PCR screening using medium-range (M-R) PCR, with primers located >600 bp away from the sgRNA cut sites (Figure 1B, C). We found several clones that harboured a LD that was not detected using S-R primers (Figure 1B, C). Indeed, multiple deleted alleles not detected using S-R PCR were observed in a pool of targeted and selected mESCs (Figure 1B). Out of a total of 84 clones that were assigned a homozygous knock-out or wild type genotype based on S-R PCR, 13 (15%) harboured a LD on one allele only detected by M-R PCR (Figure 1B, D). Five clones were further analysed using Sanger sequencing which confirmed bona fide LDs on one allele (Figure 1C). The deletions spanned 300–600 bp from either of the sgRNA cut sites at each of the three distinct genomic sites. Interestingly, one clone contained a secondary deletion upstream from the original cut site that removed one of the S-R PCR primer binding sites (Figure 1C, *, mid panel). Longer-range (L-R) PCR amplifying 5.5 kb fragments revealed even larger LDs spanning up to 2.7 kb away from sgRNA cut sites in pools of selected cells (Figure 1C, light blue dashed lines).

Importantly, PCR and sequencing based approaches may be impacted by biases including over amplification of shorter alleles containing LDs. To accurately quantify LDs in a Cas9^D10A^ nickase-targeted cell population without amplification bias, we utilized droplet digital PCR (ddPCR). Using ddPCR amplicons spaced at 500 bp, 1 kb and 3 kb intervals away from 4× sgRNA cut sites, we found that relative target DNA concentration was significantly reduced in pools of selected cells compared to non-targeting controls (Figure 1E). These findings reveal that LDs are readily detectable in pools of targeted cells irrespective of biases due to PCR primer position or design.

### Cas9^D10A^ nickase generates larger deletions at a similar frequency to Cas9 nuclease

**Figure 2.**
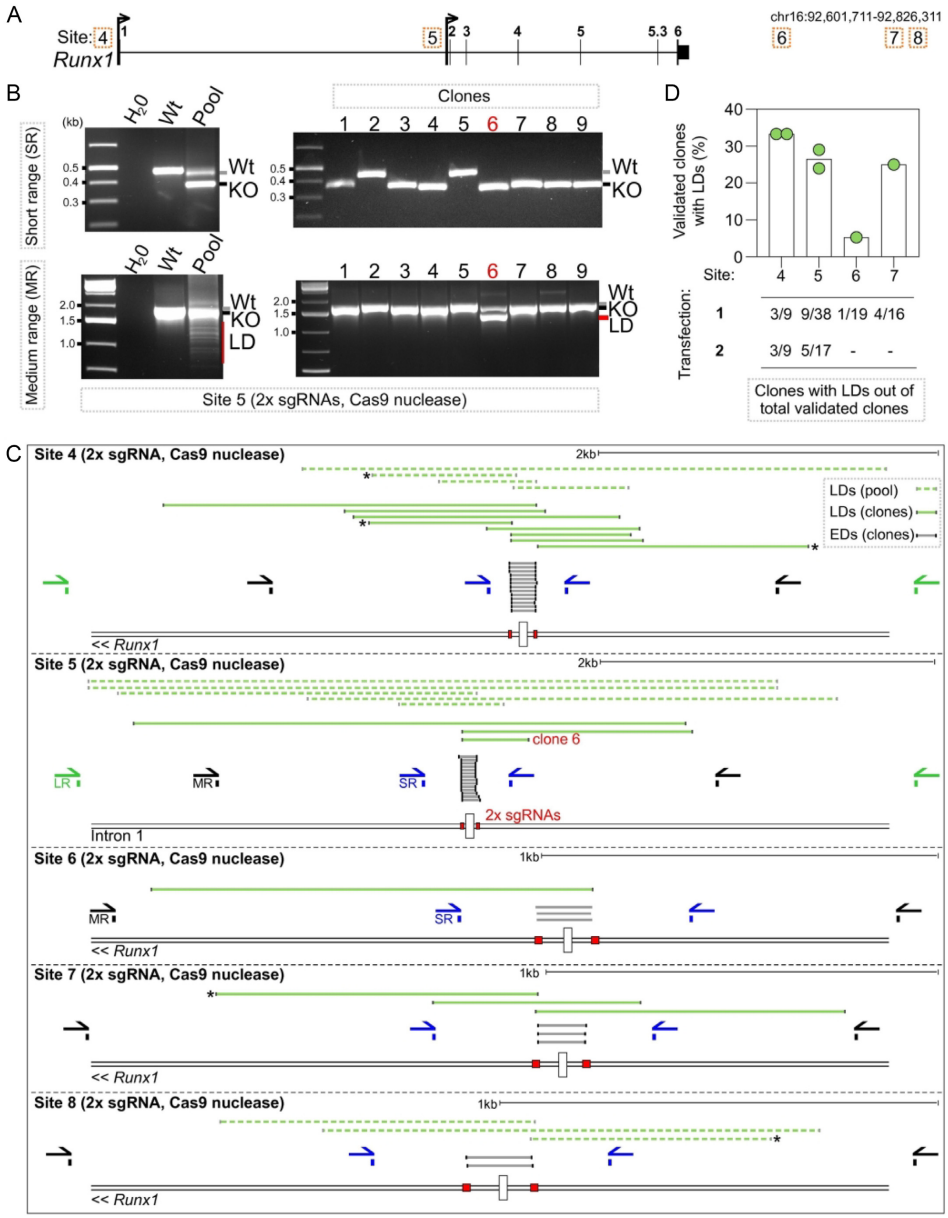
Frequency of larger deletions when genome editing with Cas9 nuclease. (**A**) Locus maps of CRISPR/Cas9 nuclease strategies to delete Sites 4–8, corresponding to transcription factor binding sites at the *Runx1* locus. (**B**) Gel images showing PCR amplification of gDNA isolated from a pool of selected cells (left-hand gels) or isolated clones (right-hand gels) targeted with Cas9 nuclease at Site 5. PCR screening was performed with SR primers (top gels) and MR primers (bottom gels). Wt next to the gel image indicates the size of the wild type allele, KO indicates the size of alleles harbouring the expected deletion, and LD indicates the size of alleles identified harbouring larger deletions. (**C**) Schematic showing the positions of S-R PCR primers (SR blue), M-R PCR primers (MR, black), L-R PCR primers (LR, green), sgRNAs (red boxes), and LDs isolated from clones (dark green lines) and pools of cells (light green dashed lines). (**D**) Quantification of clone frequencies with homozygous wild type or knock-out genotypes by S-R PCR (validated clones) that contained a LD on one allele only detected by medium-range or longer-range PCR (*n* = 1–2 independent transfections per site, each dot is one independent experiment).

To compare the occurrence of LDs between Cas9^D10A^ nickase and Cas9 nuclease, we targeted five further sites (Sites 4–8) at the *Runx1* locus using two sgRNAs (2x sgRNAs) to delete 100–150 bp per site (Figure 2A). Out of a total of 506 clones analysed by S-R PCR, 13% and 46% of individual clones targeted using Cas9 nuclease were assigned homozygous wild type or knock-out genotypes, respectively (Figure 2B, C). However, M-R and L-R PCR analysis of several apparent homozygous knock-out or wild type clones revealed that on average 23% (25/108) of these contained a LD on one allele (Compare Figure 2B, D with Figure 1C, D). Sanger sequencing of PCR products amplified from fifteen of these clones confirmed that they harboured alleles carrying LDs that abolished primer binding sites, spanning up to 2 kb away from sgRNA target sites (Figure 2C). M-R and L-R PCR analysis of pools of selected cells targeted with Cas9 nuclease revealed similar LDs to those seen at Sites 1–3 targeted with Cas9^D10A^ nickase (Figure 2C).

### Larger deletions are generated in a variety of genome editing contexts

It has been suggested that previous genetic modification employing the CRISPR/Cas9 system may select for cells with defective p53-mediated DNA repair responses (49,50). We next investigated whether previous exposure to CRISPR/Cas9 might explain the LDs that were induced by Cas9^D10A^ nickase cleavage, as the E14-TG2a-RV mESC line used had previously been targeted with Cas9. Using the same strategy as above we found that targeting *Runx1* Site 1 with Cas9^D10A^ nickase in wild type parental E14-TG2a mESCs (that had not previously undergone genome editing) generated alleles with similar LDs to those seen in cells that were previously exposed to Cas9 (Compare Supplementary Figure S2A, with Figure 1C).

To assess whether mESCs are especially susceptible to Cas9 nickase-induced LDs or whether this occurs also in other cell types, we deleted *Runx1* Site 1 in the 416B haematopoietic progenitor cell line (28) using a 4× sgRNA CRISPR/Cas9^D10A^ nickase approach (Supplementary Figure S2A). M-R PCR on gDNA isolated from a pool of electroporated (GFP-positive) 416B cells revealed shorter than expected amplicons indicative of LDs that were not detected with S-R PCR (Supplementary Figure S2A). Sanger sequencing of isolated PCR products again confirmed that the shorter PCR amplicons corresponded to LDs up to 980 bp that destroyed one of the S-R primer binding sites (Supplementary Figure S2A).

**Figure 3.**
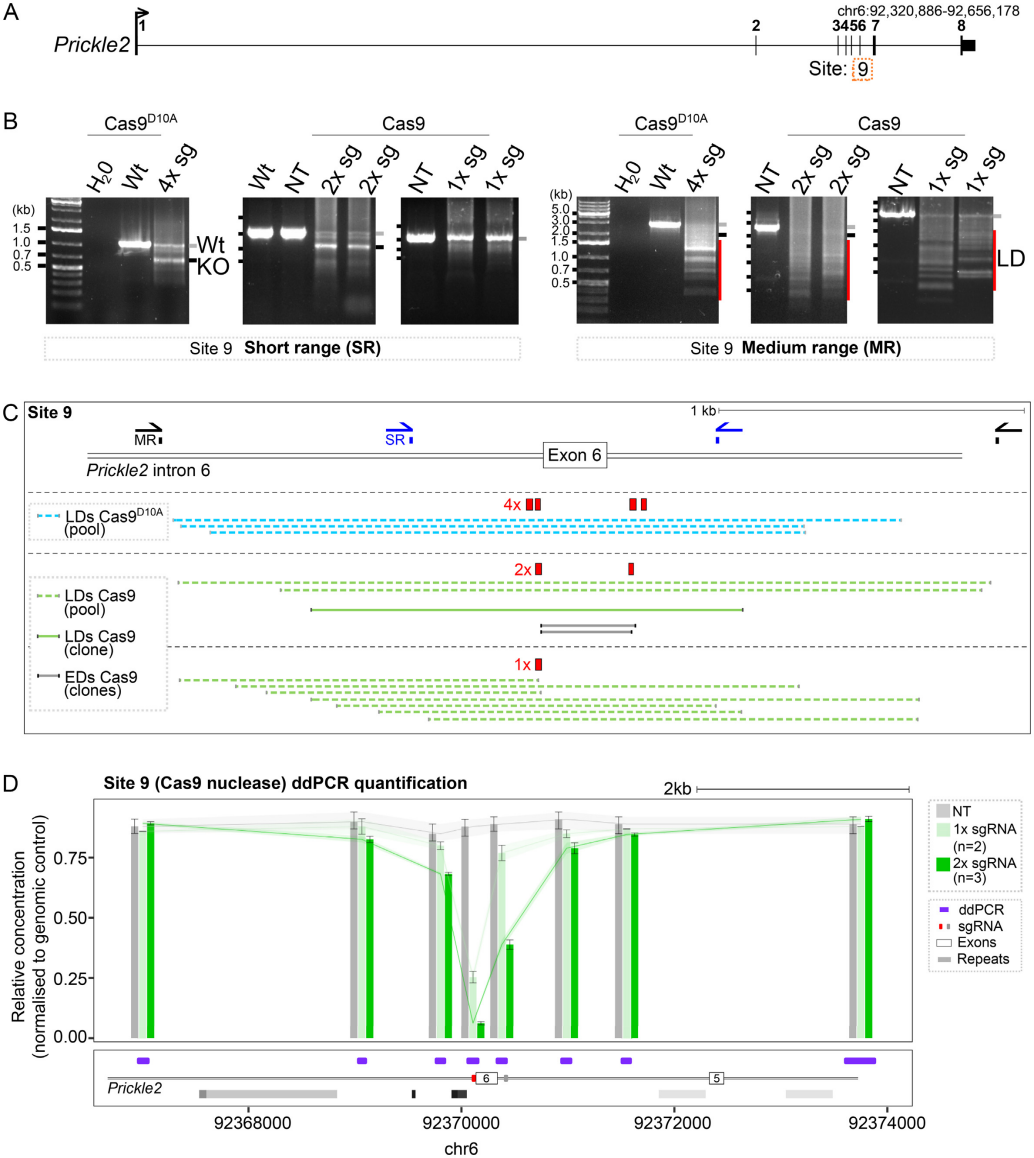
Larger deletions are generated in a variety of genome-editing contexts. (**A**) Locus schematic showing Site 9 (*Prickle2* exon 6) on mouse chromosome 6. (**B**) Gel images showing PCR amplification products from gDNA harvested from a pool of transfected cells targeted using the CRISPR/Cas9 strategies indicated. Left gel images correspond to SR primers and right gel images correspond to MR primers. Wt and a grey line next to the gel image indicates the size of the wild type allele, KO and a black line indicates the size of alleles harbouring the expected deletion (based on the location of 2× or 4× sgRNAs), and LD and a red line indicates the size of alleles identified harbouring LDs. (**C**) Schematic showing the 4× sgRNA CRISPR/Cas9^D10A^ nickase, 1× and 2× sgRNA Cas9 nuclease strategies targeting *Prickle2* exon 6. Sequenced PCR products amplified from pools of cells (light blue and light green dashed lines) and one isolated clone (dark green line). Mapped deletions of expected size (EDs) based on the location of the 2× sgRNA cut sites are shown (grey lines). (**D**) ddPCR quantification of deletions targeting exon 6 with Cas9 nuclease and 1x sgRNA (red box) or 2× sgRNAs (red and grey boxes). Each bar represents the mean ±95% confidence interval. mESCs were targeted with 1× sgRNA (light green bars, *n* = 2), 2× sgRNA (dark green bars, *n* = 3) and non-targeting control (grey bar).

As all targeted sites were located in *Runx1*, we investigated whether LDs induced by Cas9 could reflect a locus-specific feature of this gene that might not be applicable to other loci. *RUNX1* is frequently translocated in acute-myeloid leukaemia (51) and has previously been shown to be exquisitely susceptible to DSBs (52). To explore this possibility, we targeted a gene on another chromosome that has not been associated with DSBs (52) (*Prickle2* on chromosome 6). We designed a 4× sgRNA CRISPR/Cas9^D10A^ nickase and 2x sgRNA Cas9 nuclease strategy to delete 300 bp corresponding to exon 6 (Site 9) (53) (Figure 3A). PCR screening of gDNA from pools of transfected and selected E14-TG2a mESCs revealed smaller than expected PCR products that were only detected using M-R primers and were indicative of LDs when using either Cas9 nuclease or Cas9^D10A^ nickase (Figure 3B). M-R PCR screening of isolated mESC clones targeted using 2x sgRNA and Cas9 nuclease revealed a similar frequency of clones (6/17) harbouring LDs as clones targeted at *Runx1*. Sanger sequencing of M-R PCR products from isolated clones and pools of cells revealed deletions spanning up to 1.2 kb in *Prickle 2* beyond expected cut sites that abolished S-R primer binding sites (Figure 3C).

It has previously been suggested that end resection may be favoured when two DSBs are located in close proximity to each other (54), which might increase the likelihood of LD formation. However, when generating two proximal DSBs in mESCs, five alleles containing LDs spanned from only one of the two sgRNA target sites (Figure 2C, starred alleles) suggesting that either one DSB was repaired to outside of the LD in these instances, or that a single DSB is sufficient for LD formation. To examine the ability for a single DSB to generate LDs, Site 9 at *Prickle2* and Site 7 at *Runx1* were targeted with just one sgRNA each. M-R PCR and sequencing identified LDs at both sites (Figure 3B, C, Supplementary Figure S2B), implying that LD formation is independent of two adjacent DSBs. Quantitatively, ddPCR in pools of selected cells showed a slight increase in LDs when cells were targeted using 2× instead of 1× sgRNA and Cas9 nuclease (Figure 3D). Collectively, the above results show that LDs are formed in several different Cas9-mediated genome editing scenarios in cultured cells.

### Cas9-induced larger deletions also occur when genome editing *in vivo* in mouse embryos

**Figure 4.**
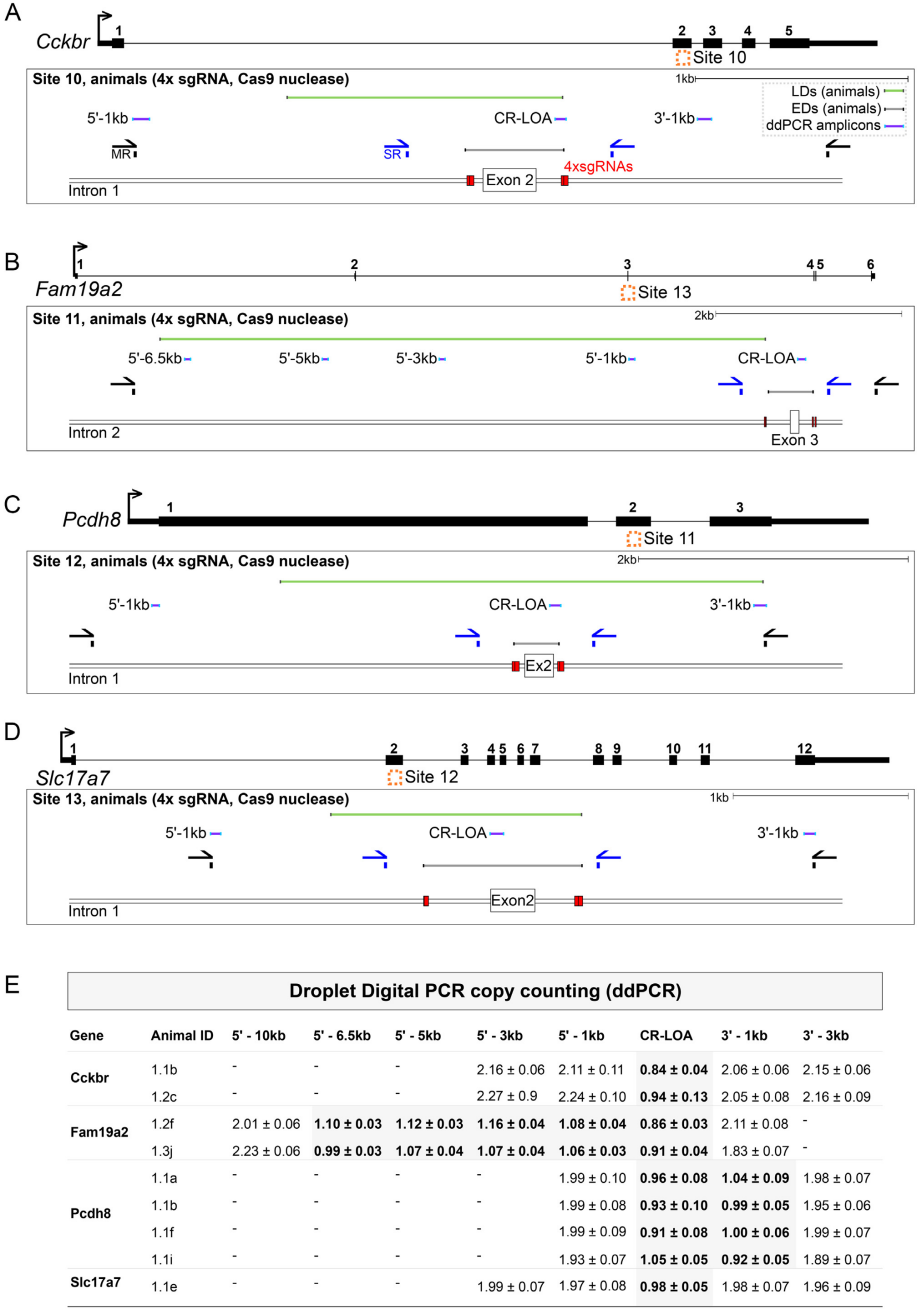
Larger deletions when genome editing in mouse embryos. (**A**–**D**) Locus maps of CRISPR/Cas9 strategies to delete Sites 10–13, corresponding to the genes *Cckbr, Fam19a2, Pcdh8* and *Slc17a7* respectively. Schematics show the positions of S-R PCR primers (SR, blue), M-R PCR primers (MR, black), sgRNAs (red boxes), ddPCR amplicons (purple lines) and LDs (green lines). (**E**) Copy counting results from ddPCR experiments. Assays against the wild type genomic sequence were designed in the critical region (CR-LOA) and at 1–3 kb intervals in the 5′ or 3′ direction distal to sgRNA cut sites (e.g. a 5′-1 kb amplicon is located 1 kb in the 5′ direction of the sgRNA cut sites). Each row corresponds to an animal where no deletion was detected by S-R PCR.

We next extended the study to the generation of Cas9-induced deletions with paired sgRNAs *in vivo* (International Mouse Phenotyping Consortium, (8)). We surveyed 32 projects aiming to create null alleles where the transmission of a deletion of an expected size had been detected by S-R PCR in the G1 generation, demonstrating sgRNA efficiency. We further validated the positive G1 animals using copy-counting of the deleted fragment by ddPCR. We ran in parallel a small number of control littermates where S-R PCR had not identified any deletion (Supplementary Table S3, Figure 4). We found control animals (without deletion detected by the S-R PCR assay) that nevertheless showed the loss of one copy of the targeted locus by ddPCR in seven out of the 32 surveyed projects. We mapped the extent of the deletion in these animals by running copy counting assays at regular intervals upstream and downstream of the intended deletion interval. Thus, we narrowed down the deletion span in these animals to a 1 kb window to each side of the paired sgRNA target sites (Figure 4). We amplified by PCR and sequenced seven of these genomic loci (Sites 10–16, targeting *Cckbr, Fam19a2, Pcdh8, Slc17a*7, *Elavl4, Scn11a* and *Trpm2* respectively), and found LDs that destroyed at least one S-R PCR primer binding site and extended up to an additional 7 kb from the intended sgRNA cut sites (Figure 4, Supplementary Figure S3).

### Microhomologies consistent with MMEJ are prevalent at larger deletions

**Figure 5.**
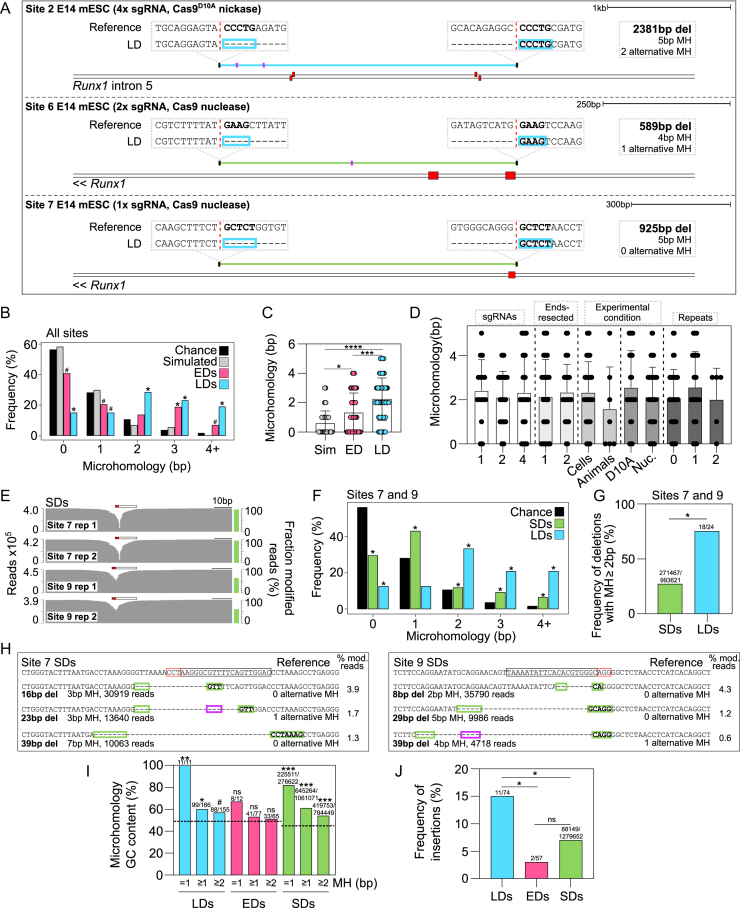
Microhomologies consistent with MMEJ are prevalent at Cas9-induced larger deletions. (**A**) Examples of LDs (blue and green lines) with microhomologies and corresponding reference sequences shown (mm9). Sequences outlined with blue boxes represent microhomologies. Red dashed vertical lines represent the exact breakpoint junctions in the repaired alleles and sgRNAs are shown (red boxes). Total deletion size, microhomology amount, and number of alternative (more proximal) microhomologies are shown (pink lines in deleted sequence). (**B**) Frequency distribution histogram of microhomologies at 74 LD breakpoint junctions (LDs) across 16 sites, 59 EDs across 16 sites (EDs), 74 simulated deletions (Simulated), and the chance expectation of finding at two locations a *k*-mer of a given length (Chance) (χ^2^ test, *, *P*< 6^−7^, #, *P*< 0.02). (**C**) Microhomology at 74 LDs compared to 59 EDs, and 74 simulated deletions (Sim) (two-tailed Kruskal–Wallis test, ****, *P*<0.0001, ***, *P* = 0.0007, *, *P* = 0.0105). (**D**) Comparison of microhomology at LDs generated with one, two or four sgRNAs, with ends resected in one or two directions, generated under different experimental conditions, or intersecting with 0, 1 or 2 repeat elements (two-tailed Kruskal–Wallis test, *P*> 0.9999). (**E**) Short-amplicon sequencing from pools of mESCs targeted using one sgRNA. Fraction of modified reads and read counts are shown. Protospacer (black outlined bar) and PAM (red outlined bar) are indicated. (**F**) Microhomology quantification in 24 LDs and all SDs mapped at Sites 7 and 9 compared to the chance expectation of finding a *k*-mer of a given length (χ^2^ test, *, *P*< 0.0003). (**G**) Quantification of deletions containing microhomology ≥2 bp in all SDs and LDs generated at Sites 7 and 9 using one sgRNA (χ^2^ test, *, *P* = 5.4^−7^). (**H**) Reference sequence and Cas9-induced deletion alleles containing significant microhomologies at their breakpoints. The total number of reads and the percentage of modified reads is shown. Protospacer (black outlined bar) and PAM (red outlined bar) are indicated. Short microhomologies that abut the deletion (green boxes) and alternative microhomologies located within the deleted region (pink boxes) are shown. (**I**) Quantification of microhomology GC base pair content in microhomologies of different lengths at all LDs, EDs, and SDs at Site 7 and 9. The expected background GC base pair content is shown as a black dashed line. (χ^2^ test, ns, *P*> 0.2, #, *P* = 0.059, *, *P*< 0.01, **, *P*< 0.001, ***, *P*< 10^−10^). (J) The number of total LDs, total EDs and SDs at Sites 7 and 9 containing a short insertion (χ^2^ test, *, *P*< 0.003, ns, *P* = 0.2395).

We next explored whether the new DNA sequences that were created after DNA breaks could inform on potential DNA repair mechanisms associated with LDs. Microhomologies of 2–5 bp in length were found at 52 out of 74 (70%) of the LD breakpoint junctions we identified (Figure 5A; Supplementary Tables S4 and S5). Homologous base pair scoring identified significantly more microhomology at LDs compared to simulated LDs, microhomology expected by chance for a *k*-mer of a given length, and microhomology found at EDs (Figure 5B, C). LDs contained microhomologies irrespective of whether LDs were generated with single or multiple sgRNAs, exhibited DNA end-resection at one or two adjacent DSBs, were generated *in vitro* or *in vivo*, by Cas9^D10A^ nickase or Cas9 nuclease (Figure 5D). There was no difference between the length or frequency of microhomologies found at LD breakpoints associated with zero, one or two annotated repeats (Figure 5D), nor were annotated repeats enriched at LD breakpoint junctions (Supplementary Figure S4). In addition to our own data, we analysed 69 Cas9-induced LDs from the literature that were previously generated by single or pairs of sgRNAs (8,19–24). These 69 distinct LDs also contained a significant over-representation of microhomologies compared to the chance expectation or simulated deletions (Supplementary Figure S5; Supplementary Table S5). Altogether, these data suggest that MMEJ is active during the repair of LD alleles, as MMEJ depends on short (<20 bp) microhomologies that are shared between both breakpoints, with some tolerance for mismatches (7).

MMEJ was previously implicated in the repair of Cas9-induced DSBs at shorter deletion alleles (SDs) of <60 bp (15,39,55–61). To directly compare the prevalence of microhomologies at Cas9-induced SDs with LDs, we performed short-amplicon deep sequencing after targeting two different chromosomes with 1x sgRNA each (Figure 5E). LDs at Sites 7 and 9 (characterised in Figure 2 and Figure 3) were significantly enriched for microhomologies compared to SDs quantified at the same sites (Figure 5F, G). Still, microhomologies were significantly over-represented at SDs compared to the chance expectation of two sequences containing a *k*-mer of a given length (Figure 5F, H). MMEJ has previously been shown to favour thermostable microhomologies with elevated GC content (46,55). Microhomologies at all Cas9-induced LDs and SDs at Sites 7 and 9 were both significantly enriched for GC base pairing compared to background (Figure 5I, Supplementary Figure S6), while microhomologies across all EDs were observably but not significantly enriched (Figure 5I). Interestingly, GC bases were always the most enriched in microhomologies of 1 bp, compared to longer microhomologies (Figure 5I). MMEJ is also known to frequently generate small non-templated insertions (62,63). In line with this, Cas9-induced LDs were enriched for small insertions compared to EDs and SDs (Figure 5J, (Supplementary Figure S7). Collectively these data show that the majority of larger Cas9-induced deletions contain microhomologies consistent with MMEJ at their breakpoints.

### Larger deletion distribution is dependent on proximity to sgRNAs and cannot be predicted by microhomology sequences alone

**Figure 6.**
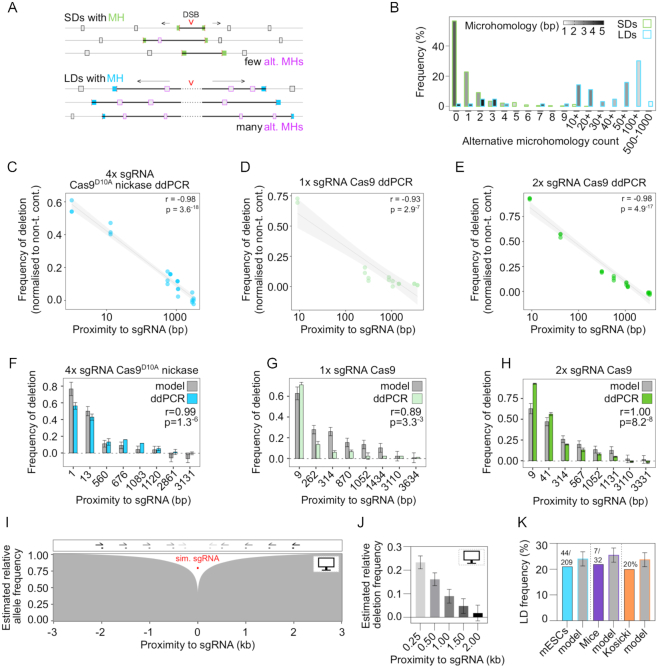
Larger deletion breakpoints do not occur at proximal microhomology sequences but are dependent on proximity to sgRNAs. (**A**) Schematic representation of LDs and SDs undergoing end-resection and bypassing alternative more proximal microhomologies during DNA repair. SD microhomologies are shown in green, LD microhomologies are shown in blue, and alternative microhomologies are indicated by pink boxes. The sequence included in the deletion is shown as a bold black line. (**B**) Quantification of alternative microhomologies that were found in the deleted sequences at Sites 7 and 9 SDs and across all LDs with microhomologies at their breakpoints. The colour gradient represents the mean microhomology score of all deletions within each bin. (**C**–**E**) Correlation between frequency of deletion determined by ddPCR and sgRNA proximity. Pearson correlation *r* and *P* values are indicated and a linear regression with 95% confidence interval is shown. (**F**–**H**) Deletion frequencies of real ddPCR data and model estimates with Pearson correlation *r* and *P* values indicated. (**I**) Model estimate of deletion frequency over a 6 kb window around a simulated sgRNA cut site with simulated PCR primers indicated as grey to black half arrows above the plot. (**J**) Relative predicted deletion frequencies at each of the simulated primer sites with 95% confidence intervals indicated. (**K**) Comparison between estimated and empirically determined deletion frequencies in two of our own independent data sets and one recent experiment reported in the literature (17).

Recent work has suggested that DNA repair outcomes are predictable at Cas9-induced DSBs based on the presence of microhomologies in cut site-proximal DNA sequences (39,55–58). We asked whether the distribution of LDs was similarly dependent on the proximity of microhomologies to cut sites. At LDs, deletion size was independent of microhomology length, unlike at SDs (Supplementary Figure S8A). For all but one LD, the intervening sequence between deletion ends and sgRNA cut sites contained several alternative (more proximal) microhomologies that were bypassed during repair (median = 49, Figure 6A, B). In contrast, microhomologies used for repair at SDs were predominantly (but not exclusively) the most proximal to the cut site (Figure 6A, B, Figure 5H). The number of alternative microhomologies present in the deleted sequence was dependent on deletion length and microhomology length (Supplementary Figure S8B, C, D), reflecting the random distribution of microhomology sequences throughout the genome. Together this indicates that in contrast to SDs, LDs are not repaired to the closest microhomology.

Given the fact that LD sizes are independent of cut site proximal microhomology sequences, we examined what other factors might influence LD formation. At the population level, the distribution of deletion sizes as inferred from ddPCR was negatively correlated with proximity to sgRNA target sites (Figure 6C–E). We modelled this relationship using multiple linear regression and found that over 80% of the variance in the distribution of deletion sizes depended on proximity to sgRNAs and sgRNA cutting efficiency determined by ddPCR (Figure 6F–H, Supplementary Figure S9, adjusted *R*^2^ = 0.8275, *P* < 2^−16^). Interestingly, the model based on empirical ddPCR measurements estimated that in general 22 ± 3% of alleles were deleted 250 bp from sgRNA target sites (Figure 6I, J). This agrees with the 21% (44 out of 209) of our isolated mESC clones that harboured LDs abolishing S-R PCR primers (Figure 1D, Figure 2D, Figure 6K, mean sgRNA proximity = 243 bp). Furthermore, S-R primer binding sites with a mean sgRNA proximity of 211 bp were abolished in 22% (7 out of 32) mouse projects (Figure 4, Supplementary Figure S3, Figure 6K). A recent study also found that LDs >250 bp occurred in up to 20% of alleles (17,64) (Figure 6K).

## DISCUSSION

### Unintended larger Cas9-induced deletions are common with both Cas9 nuclease and nickases

We have characterised unintended larger than expected on-target deletions induced by CRISPR/Cas9 genome editing. These deletions were found at sixteen sites across nine gene loci on seven different chromosomes. They were not linked to previous exposure to Cas9 nor restricted to one particular cell type. The frequency of larger than expected deletions in response to Cas9-induced DSBs was in keeping with earlier reports (8,12,17–21,24–27). Notably, LDs occurred at a comparable frequency with Cas9 nuclease and Cas9^D10A^ nickases and were found *in vitro* and *in vivo*. Thus, although Cas9^D10A^ nickase is favourable over Cas9 nuclease for use in gene therapy based on its reduced off-target effects (5), it is equally prone to generating LDs. The largest unintended on-target deletion we identified spanned an additional 7 kb away from the target sgRNA sites. Often LDs coincided with the location of the PCR primer used in a particular screen, making it likely that even larger deletions are generated. In one previous study, an edited allele was shown to harbour a deletion spanning 42 kb, the largest deletion reported so far (21). We showed that LDs cannot be predicted by proximal regions of microhomology. Instead the distribution of LDs in a cell population could be modelled using computational approaches based on proximity to sgRNA cut sites and sgRNA cutting efficiency. This provides important information for the future design of genome editing experiments. Moreover, it lays the foundation to build more robust models that may be able to accurately predict LDs.

### Considerations for the use of genome editing

Failure to detect LDs could lead to the misidentification of a heterozygous deletion as a wild type or homozygous knock-out genotype and misinterpretation of experimental results. Critically, such oversight would also lead to failure to detect potential oncogenic mutations when editing for gene therapy (17,65). Of note, LDs may also be generated at off-target sites. Given the scale and frequency of potentially deleterious LDs, robust screening methods need to be employed to ensure their timely detection. To limit allelic drop-outs when using PCR-based methods, primers should be designed as far away as possible from sgRNA binding sites. Complementary screening methods can be used to increase confidence in genome editing outcomes. Compound heterozygote clones or animals (with different deletions on each allele) can be identified by a convoluted Sanger sequencing read beginning at the sgRNA cut site. Because two copies of the target region are detected, they should be less likely to contain a LD or allelic drop-out (27). Southern blotting has also been used to investigate targeted deletions and insertions by genome editing (66). However, LDs extending beyond a hybridization probe would still be undetectable and copy number analysis would need to be used to alleviate this. Whole-genome sequencing (WGS) is a robust method to detect genome editing outcomes (67) but may be prohibitively expensive for most applications of CRISPR/Cas9 as specialized mate-pair or paired-end sequencing approaches and high coverage must be used to reliably detect chromosomal rearrangements (68,69). However, DNA target capture has been used to reduce sequencing costs (59). Alternatively, fluorescence *in situ* hybridisation (FISH) (70), and chromosome conformation capture (3C)-based methods have been used to detect structural variants (71,72). In contrast to all these methods, copy number counting by ddPCR is much simpler to implement and does not require prior knowledge about the deletions being screened. It also allows quantification of the frequency of events in populations of cells with a mix of complex genotypes and distinguishes between an *iso*-allelic deletion (two copies of an identical allele that were repaired by HR), and the presence of LDs (this study and ref. (8)), which is not possible by Sanger sequencing or targeted next-generation sequencing (73).

As well as considering different methods of detection, it may be beneficial to develop methods to reduce the occurrence of LDs. It has been suggested that long-term exposure to sgRNA either through plasmid or lentivirus delivery may increase the frequency of larger indels (>6 bp) (74). Some of our experiments used plasmid delivery leading to relatively prolonged sgRNA and Cas9 expression, which might contribute to the observed high frequency of LDs. Both RNP-delivery (75) and conditional Cas9 approaches (76) limit the time that Cas9 is active within cells and, therefore, might reduce off-target effects and be favourable for gene therapy. However, we also detected LDs when using Cas9 mRNA to perform genome editing, demonstrating that plasmid exposure is not a prerequisite for larger Cas9 deletions, in line with recent results (17,24).

### Possible mechanisms for larger deletion generation

Microhomologies were significantly overrepresented at LDs irrespective of site, Cas9 used, whether targeting cultured cells or embryos, or whether DNA end-resection occurred in one or both directions. This may hint at a common molecular mechanism for LD generation downstream of DSB formation. Since we found no association between annotated repeat elements and LDs, SSA is an unlikely mechanism for LD generation. The size of LDs was also outside the normal range for NHEJ (12–16), making this an unlikely mechanism. Since we found microhomologies were significantly overrepresented at LD breakpoint junctions, this suggests that microhomologies are preferentially used as part of the repair mechanism that generates LDs, possibly through MMEJ. MMEJ has previously been implicated in targeted insertions (77–80) as well as deletions, but in previous studies was associated with smaller deletions than the LDs we observed (7,15,39,46,55–61). MMEJ has also been implicated in chromosomal translocations (7,81,82), which by their nature take place over large genomic sequence scales. A recent study found that MMEJ was most active with 5 bp microhomologies (81), which is in line with the overrepresented short microhomologies we observed at Cas9-induced LDs. Moreover, MMEJ repair is thought to favour GC base paring (46), which we also observed at Cas9-induced LDs. Together, these findings are consistent with a possible role for MMEJ in LD formation.

In the context of genome editing, what could cause cells to undergo extensive end resection at Cas9-induced DSBs? One possibility is that during the repair of a DSB, the HR pathway is compromised by both alleles being targeted by Cas9. In preparation for HR, extensive 5′ to 3′ resection occurs at DSB DNA ends, inhibiting NHEJ (83,84). However, because both alleles are likely to be targeted and cut by Cas9 concomitantly, productive HR may fail. It is possible that after extensive end resection and abortive HR, MMEJ then repairs the allele, generating LDs. Indeed, it has previously been suggested that if NHEJ fails, alternative end joining pathways such as MMEJ or SSA are favoured (61,84–86). Whether MMEJ is a back-up survival or primary DNA repair mechanism also remains uncertain (7). Future genetic studies will be needed to determine a critical dependency of Cas9-induced LDs on MMEJ.

Despite finding significant microhomologies at Cas9-induced LDs, we cannot rule out other DSB repair mechanisms also playing a role. Another possible mechanism for LD generation could involve interference with the normal functions of the NHEJ pathway by Cas9 residing on the DNA template. Cas9 has a residence time on DNA of >3 h (87), and might interfere with the normal function of the Ku70/80 heterodimer, which binds to DNA ends and is required for NHEJ (6). In support of this, it has been shown previously that inactivating the Ku80 protein favours larger deletions (7,88,89). In order to test this, a weaker binding genome-editing modality (such as hfCas9 (2)) that might interfere less with Ku binding or function could be tested for its ability to generate LDs. Alternatively, small molecule inhibition of NHEJ (90) could elucidate whether LDs are NHEJ-dependent. Gaining a full molecular understanding of LD generation may be challenging, in part because multiple repair pathways may be active at the same locus (61).

In summary, our findings emphasize the fact that larger than expected deletions are generated at a high frequency when genome editing. LD breakpoint junctions occur at regions with significant microhomology, implicating MMEJ as a possible DSB repair pathway in their formation. In contrast to SDs, the LDs cannot be predicted by proximal microhomology sequences. Instead, the distribution of LD sizes can be modelled in cell populations based on proximity to sgRNA cut sites.

## DATA AVAILABILITY

Raw fastq files have been deposited in NCBI’s Gene Expression Omnibus (41) and are accessible through GEO Series accession number GSE130621. All custom scripts are freely available on GitHub (https://github.com/d0minicO/mhscanR).

## Supplementary Material

gkz459_Supplemental_Files
