# Supplementary material for: Microhomologies are prevalent at Cas9-induced larger deletions
Source: Nucleic Acids Res. 2019 May 25;47(14):7402–17. doi: 10.1093/nar/gkz459 (PMC6698657; doi:10.1093/nar/gkz459)
Supplement: gkz459_Supplemental_Files [file gkz459_supplemental_files.pdf]

Supplementary Figure and Table legends

Supplementary Figure 1 – Overview of genome editing in mouse embryonic stem cells

A) Protocol schematic for mESC gene-editing, colony-picking, genotyping, and expansion. B) An example short-range PCR genotyping gel result from one experiment targeting Site 1 in mESCs with CRISPR/Cas9^{D10A} nickases. The wildtype amplicon size is 2 kb, and the knockout amplicon size is 500 bp. C) Genotype frequencies from three independent experiments at each Site 1-3 determined by short-range PCR screening. D) Average genotyping results from all of the three experiments at Sites 1-3. E) Example Sanger sequencing traces from PCR products amplified from clones that were identified as homozygous knock-out clones with short-range PCR. A typical 'clean trace' read (top trace) and 'mixed trace' read (bottom trace) are shown for comparison. The site of the beginning of the 'mixed trace' is indicated with a red arrow head.

Supplementary Figure 2 – Larger deletions are generated in a variety of genome-editing contexts

A) Schematic of CRISPR/Cas9^{D10A} nickase strategy to delete Site 1 on chromosome 16 showing the positions of short-range PCR primers (SR, blue), medium-range PCR primers (MR, black), sgRNAs (red boxes), and LDs from E14-TG2a mESCs (E14, light blue dashed lines) and 416B cells (dark red dashed lines). Gel images showing PCR amplification products from gDNA harvested from a pool of transfected/electroporated cells. Site 1 was targeted in E14-TG2a mESCs that had not previously undergone CRISPR/Cas9 editing and in the 416B haematopoietic progenitor cell line. Left gel images correspond to short-range primers and right gel images correspond to medium-range primers. Wt and a grey line next to the gel image indicates the size of the wild type allele, KO and a black line indicates the size of alleles harbouring the expected deletion, and LDs and a red line indicates the size of alleles identified harbouring LDs. B) Site 7 Cas9 nuclease and 1x sgRNA targeting strategy. The sgRNA included in the 1x sgRNA transfection is shown in red. LD alleles amplified from pools of targeted cells are shown as light green dashed lines. Gel images showing PCR amplification products from pools of cells targeted using the indicated number of sgRNAs. Amplification from a pool of cells targeted with 2x sgRNA (in a separate experiment) is shown for comparison. Both the sgRNAs marked in grey and red were included in the 2x sgRNA experiment.

Supplementary Figure 3 – Larger deletions identified when genome editing *in vivo* in mouse zygotes

Locus maps of CRISPR/Cas9 strategies to delete Sites 14-16 in mouse embryos, corresponding to the genes *Elavl4*, *Scn11a*, and *Trpm2*, respectively. Schematics show the positions of short-range PCR primers (SR, blue), sgRNAs (red boxes), and larger deletions (green lines). Copy counting ddPCR amplicons are indicated as purple lines. ddPCR amplicons were designed against the wildtype sequence at the critical region to identify loss of the allele (CR-LOA) and at 1 to 3 kb intervals in the 5' or 3' direction distal to sgRNA cut sites (e.g. a 5'-1 kb amplicon is located 1 kb in the 5' direction of the sgRNA cut sites).

Supplementary Figure 4 – Quantification of repeat elements associated with larger deletions

A) Annotated repetitive elements (green boxes with black outlines) were mapped alongside LDs using the UCSC genome browser RepeatMasker. LDs were defined as having neither end (grey bars), one end (orange bars), or two ends (red bars), intersecting within 100 bp of annotated repeat elements. A secondary deletion that was upstream from the original cut site and removed one of the S-R PCR primer binding sites in a clone targeted at Site 2 was contained within a simple tandem repeat (red bar marked with *) B) Quantification of repeat intersections with 74 LDs and the same number of equally sized simulated deletions (****, $\chi 2$ test, $p < 0.0001$).

Supplementary Figure 5 – Previously published larger deletion alleles analysed for the presence of microhomologies

A) Representative previously published (Adikusuma, 2018) LD alleles (green lines) identified at two selected sites targeted with 1xsgRNA (red bars) and Cas9 nuclease. B) Selected LD alleles previously published (Adikusuma, 2018) and the amount of microhomology that could be identified. Examples of LDs with microhomologies and corresponding reference sequences shown (mm9). Sequences outlined with blue boxes and highlighted in bold represent microhomologies. Red dashed vertical lines represent the exact breakpoint junctions in the repaired alleles. Total deletion size and microhomology amount are indicated. C) Frequency distribution of microhomology amount found in 69 previously published LDs (Wang 2013, Zhou 2014, Ma 2014, Parikh 2015, Zhang 2015, Mianne 2017, Adikusuma 2018) that were analysed for the presence of microhomology, the same number of simulated deletions across the genome with the same average length as LDs (Simulated), and the expected probability of finding homology at two sites for a *k*-mer of a given length (Chance) (*, $\chi 2$ test, $p < 0.0001$). D) Quantification of the amount of microhomology identified at 69 previously published LD alleles and simulated deletions (****, Mann-Whitney test, $p < 0.0001$). All previously published LD alleles and microhomologies identified are available in Supplementary Table 5.

Supplementary Figure 6 – Shorter Cas9 deletion distribution determined by deep sequencing to calculate background GC base pair content

A) Histogram plots showing the number of deletion end points that were mapped along the entire sequenced region after targeting Sites 7 and 9 with one sgRNA each. The distribution of deletion ends approximates a normal distribution (smooth blue line) centred on the expected cut site (dashed red line). B) 93.5-94.5% of total deletion end points are contained within the amplicon region around the mean +/- 2 standard deviations (μ +/- 2σ) of deletion ends. The GC base pair content over this region was taken as the expected GC base pair content of microhomologies found if there was no GC bias.

Supplementary Figure 7 – Selected LD alleles that contained insertions and less microhomology

A) LD alleles with short insertions at Sites 1, 9 and 15. Inserted sequences are highlighted with a purple box and microhomologies are highlighted in bold and with a blue box. B) Microhomology quantification at all LDs with and without short insertions (*, Mann Whitney test, $p<0.0001$).

Supplementary Figure 8 – Distribution of microhomology sequences is dependent on microhomology length and deletion size

A) Scatter plot showing deletion size vs microhomology size at all LDs and all SDs at Sites 7 and 9. Linear regression was done using the given formula and shown on the plot with 95% confidence intervals. R^2 and p values are indicated above the plot with Pearson correlation r and p values. B) Scatter plot of alternative microhomology count vs microhomology size at all LDs and all SDs at Sites 7 and 9 with microhomology. Linear regression was done using the given formula and shown on the plot with 95% confidence intervals. R^2 and p values are indicated above the plot with Pearson correlation r and p values. C) Scatter plot of alternative microhomology count vs deletion size at all LDs and all SDs at Sites 7 and 9 with microhomology. The red line (y=x/4) indicates the chance of finding one nucleotide in a stretch of DNA with evenly distributed nucleotides of length x. Linear regression was done using the given formula and is shown on the plot with 95% confidence intervals. R^2 and p values are indicated above the plot with Pearson correlation r and p values. D) Multiple linear regression was done using the given formula with adjusted R^2 with p values, and residuals with p values indicated. The higher R^2 values in (D) compared with (B and C) indicates that alternative microhomology count is dependent on the combination of both deletion size and microhomology size.

Supplementary Figure 9 – Multiple linear regression model of the distribution of Cas9-induced larger deletion sizes

A) Histogram of residuals for the model (where residual = observed value - predicted value). B) Normal Q-Q plot for the model to check that residuals are normally distributed (which is an assumption of the linear regression model). C) Summary of multiple linear regression using the given formula with adjusted R^2, p values, and residuals with p values indicated.

Supplementary Table 1 – single guide RNAs used in the study

Supplementary Table 2 – Primers used in the study

Supplementary Table 3 - Summary of gene editing experiments in mouse zygotes in vivo

Supplementary Table 4 - Summary of deletions analysed per site

Supplementary Table 5 - Mapped deletion breakpoint sequences

Supplementary Figure 1

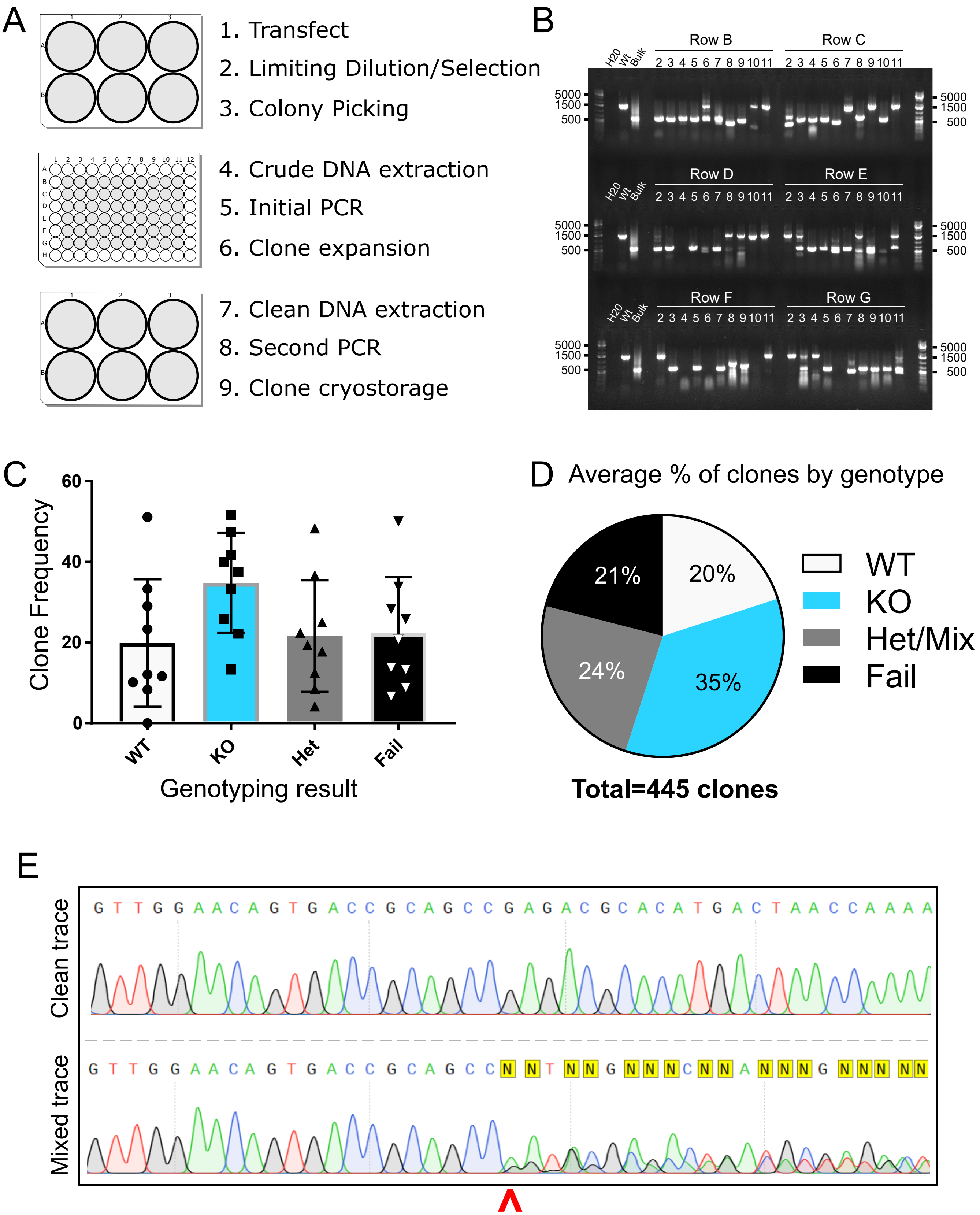

Supplementary Figure 2

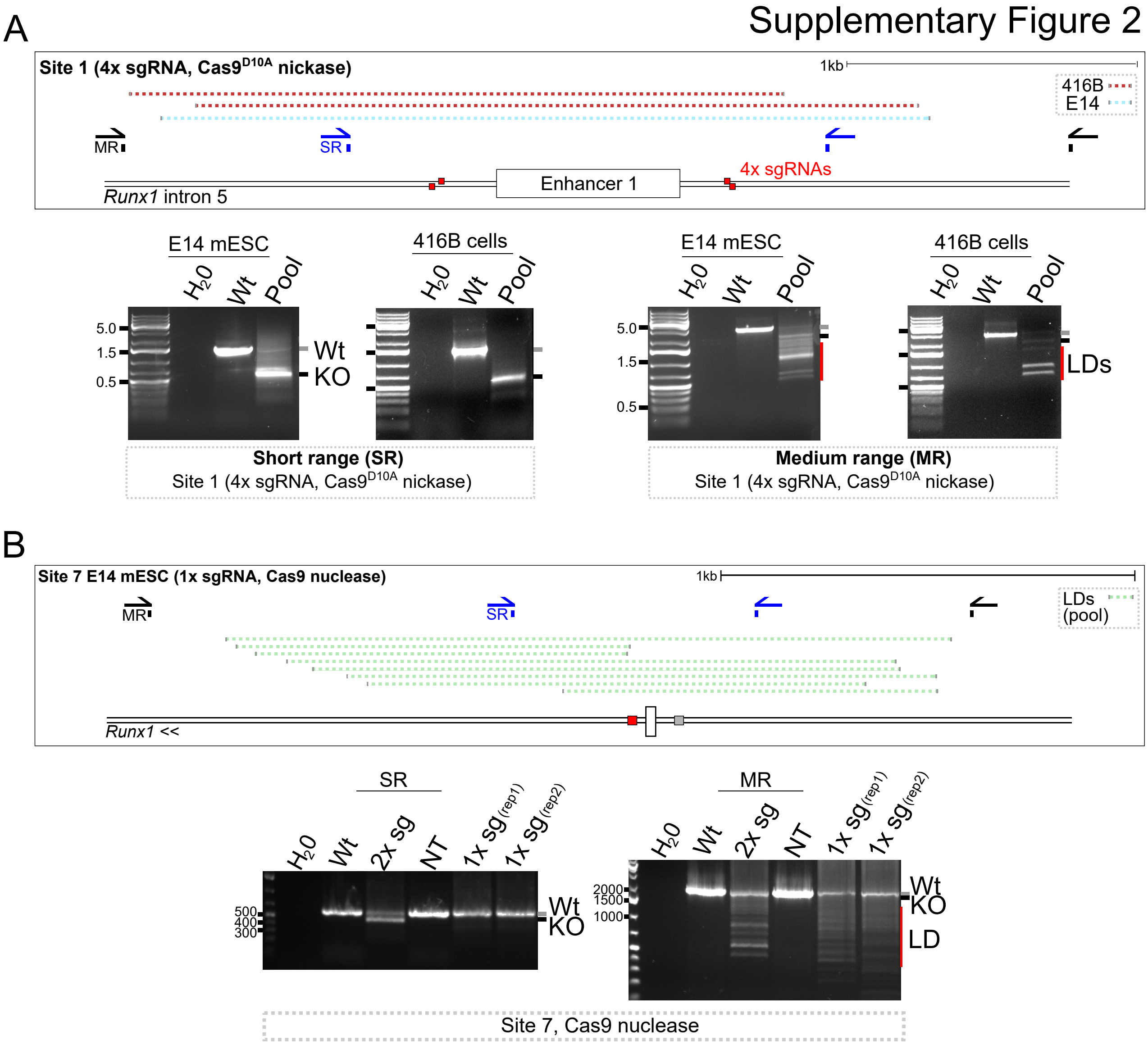

Supplementary Figure 3

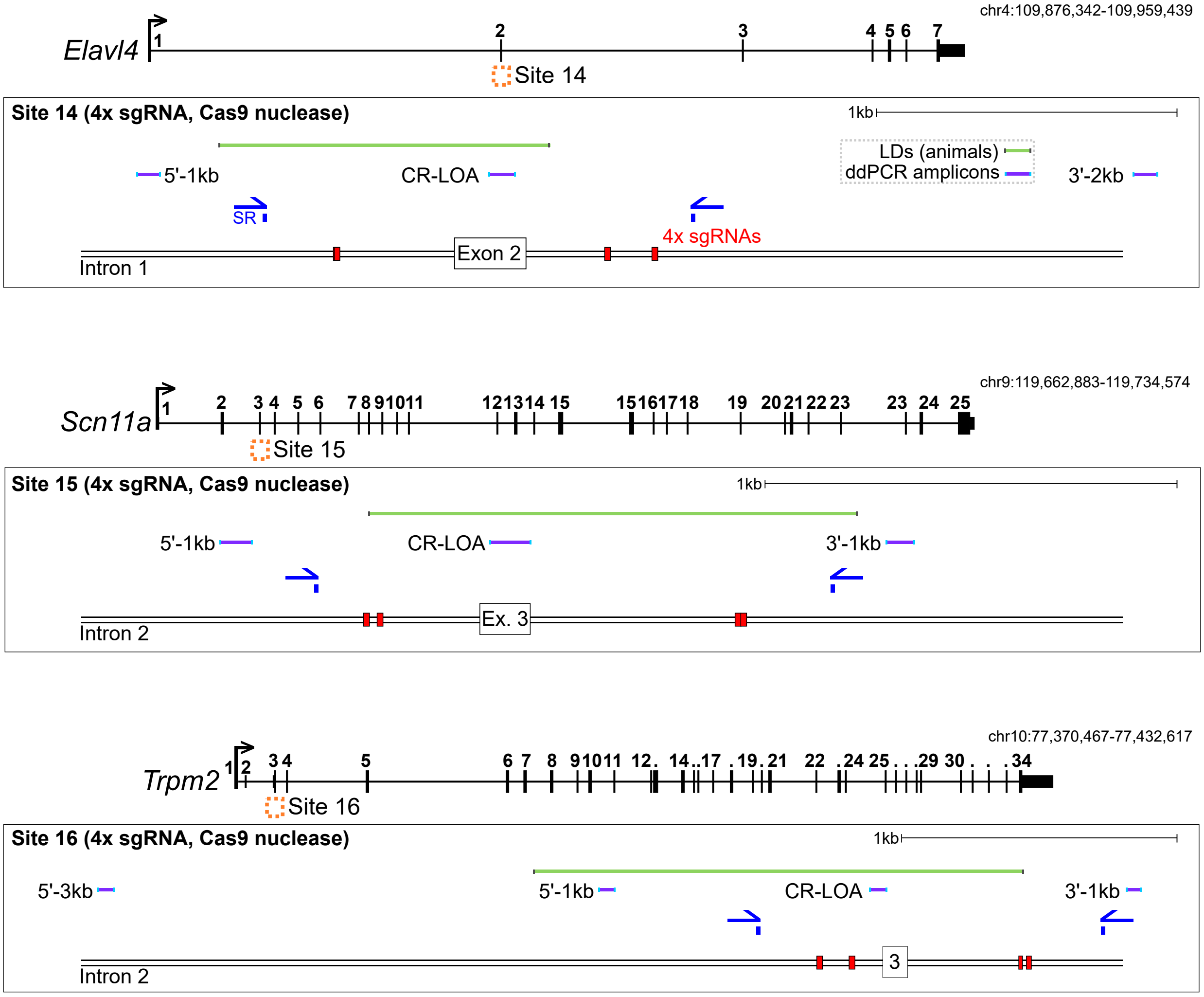

Supplementary Figure 4

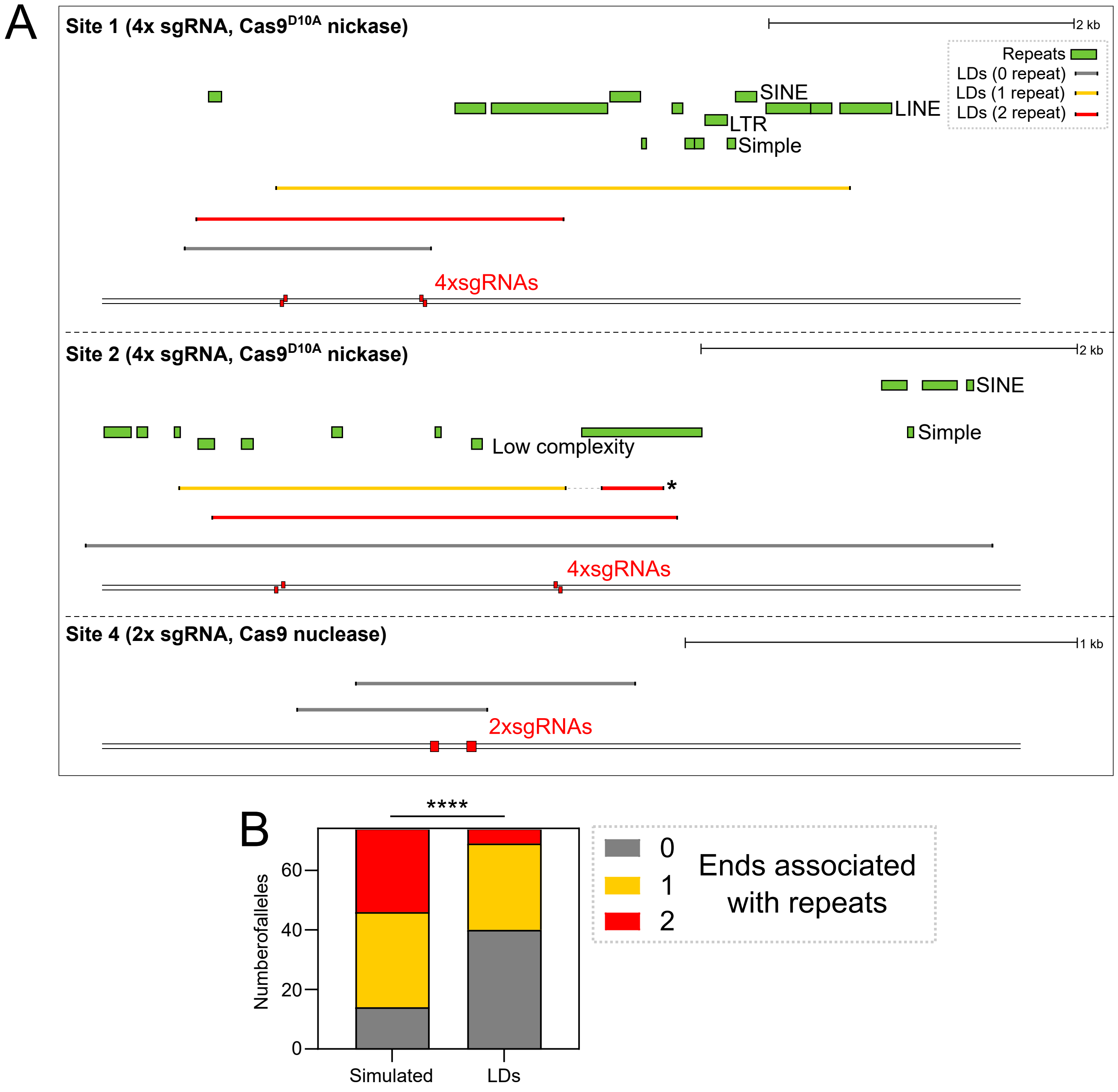

Supplementary Figure 5

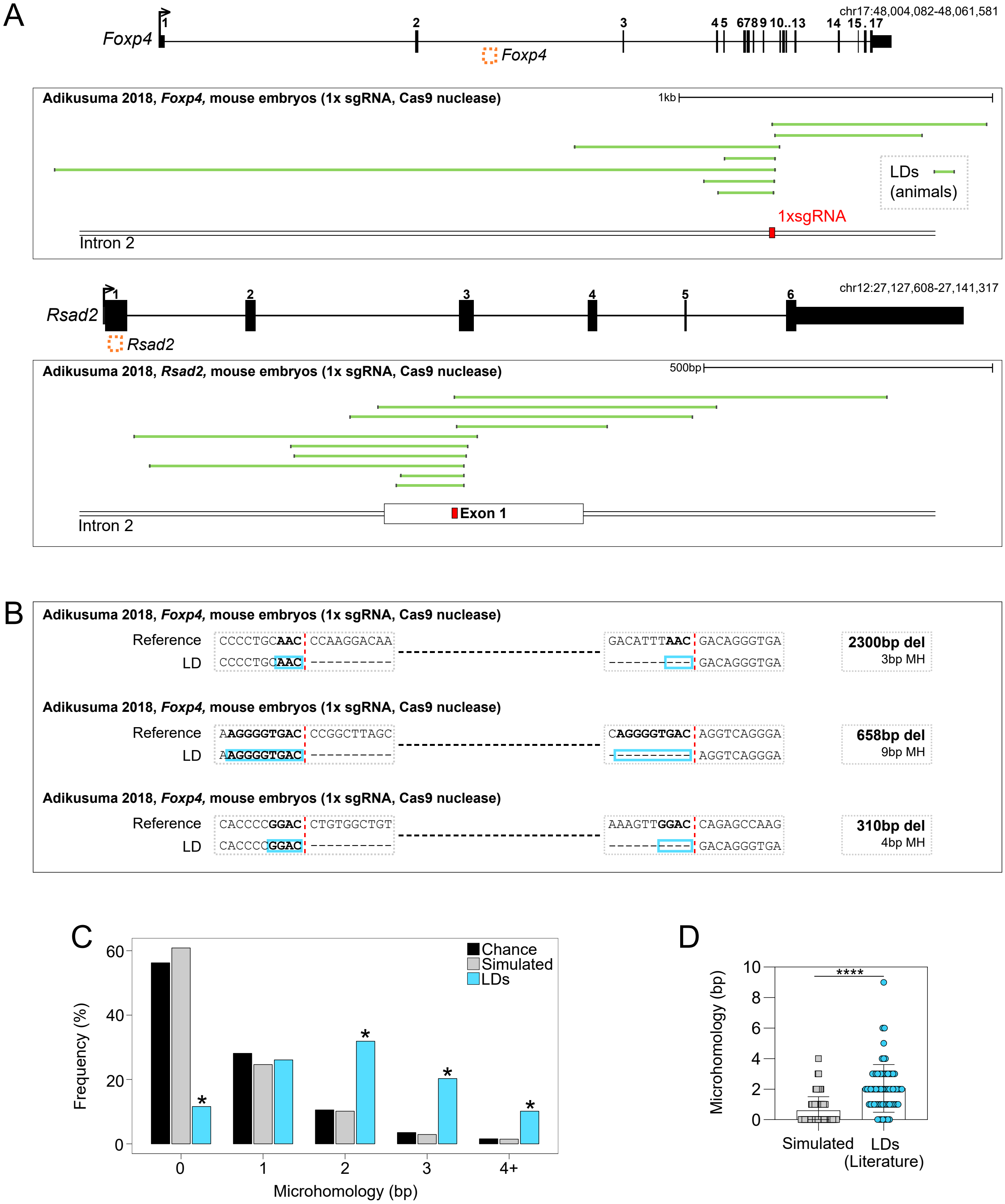

Supplementary Figure 6

A

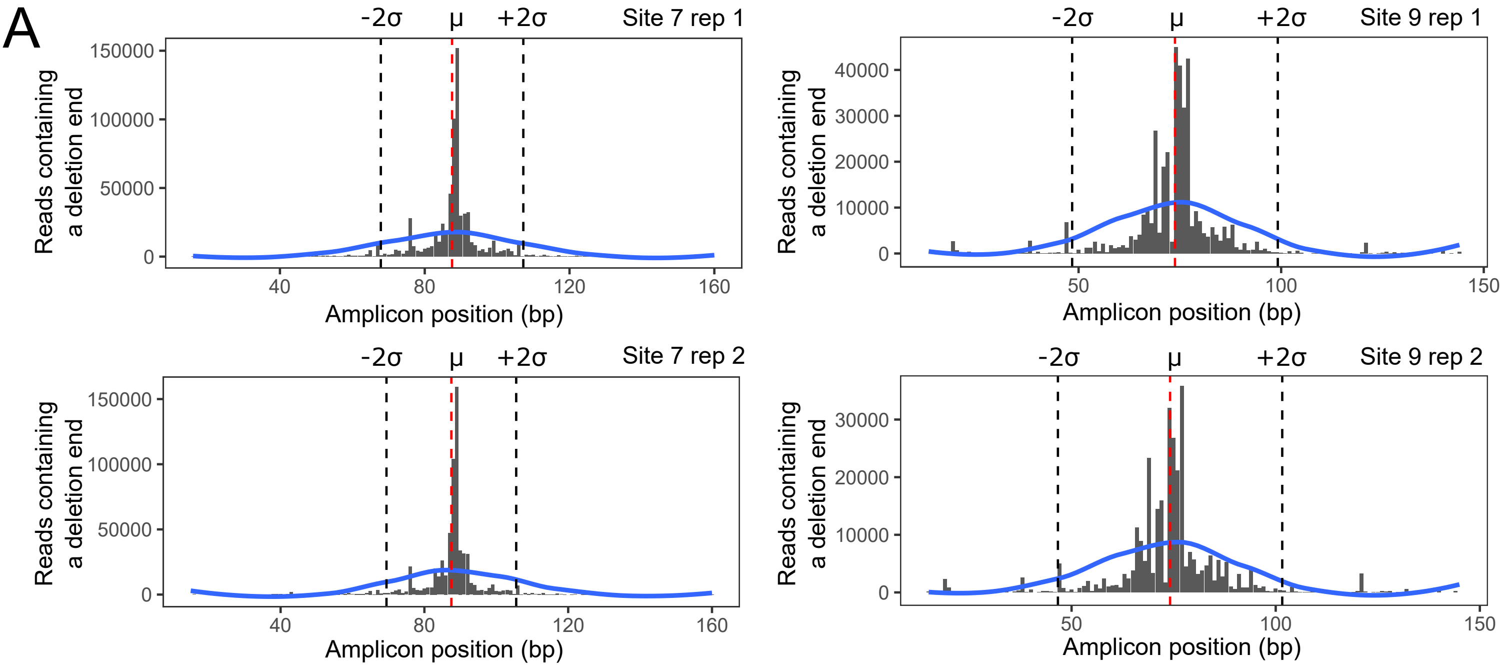

B

Sample	Mean (μ)	S.d. (σ)	% of Reads (μ +/- 2σ)	GC% (μ +/- 2σ)
Site 7 rep 1	87.5	9.0	94.2	43.2
Site 7 rep 2	87.6	9.8	94.3	46.3
Site 9 rep 1	73.8	12.7	93.5	48.1
Site 9 rep 2	74.1	13.7	94.5	48.2

Supplementary Figure 7

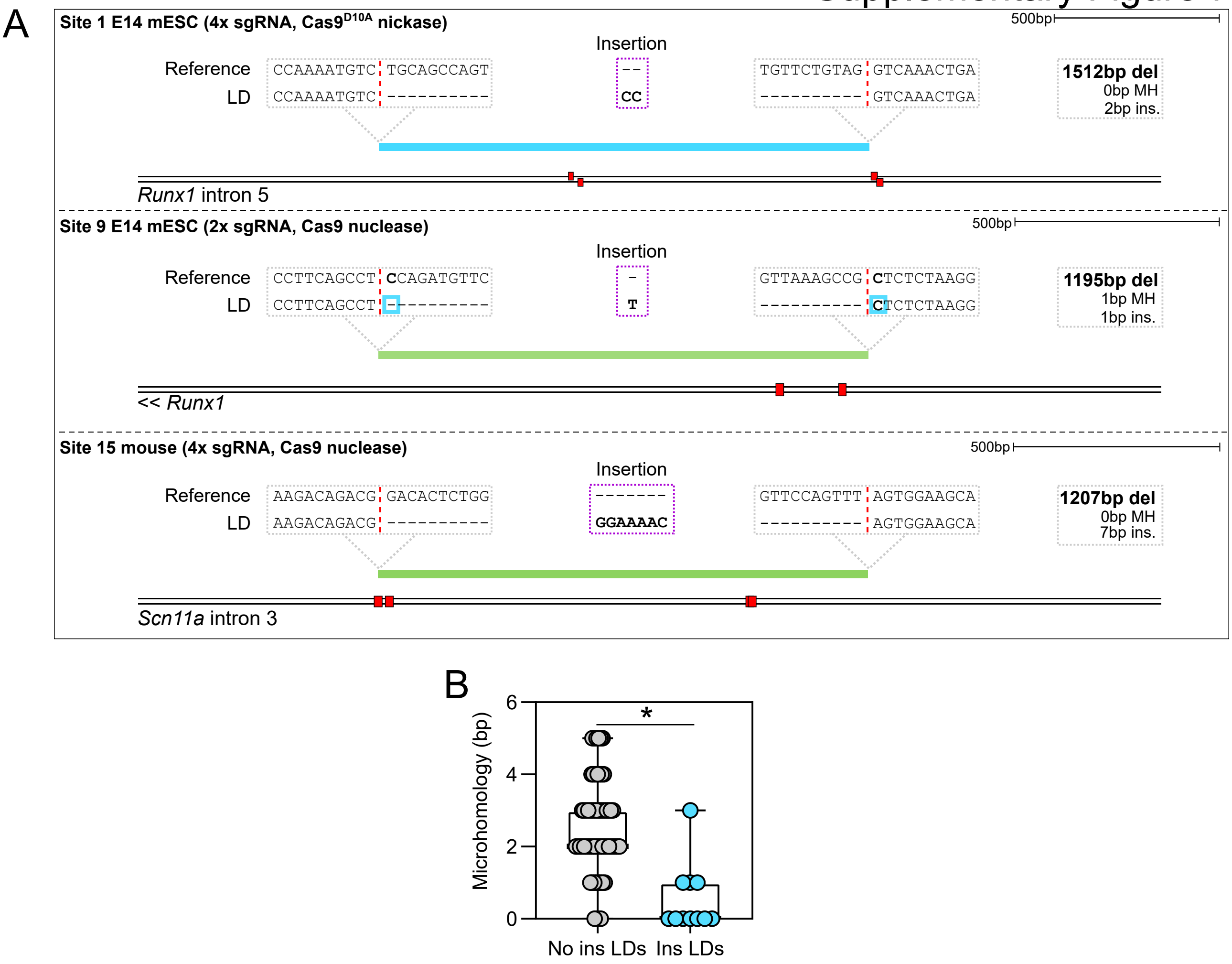

Supplementary Figure 8

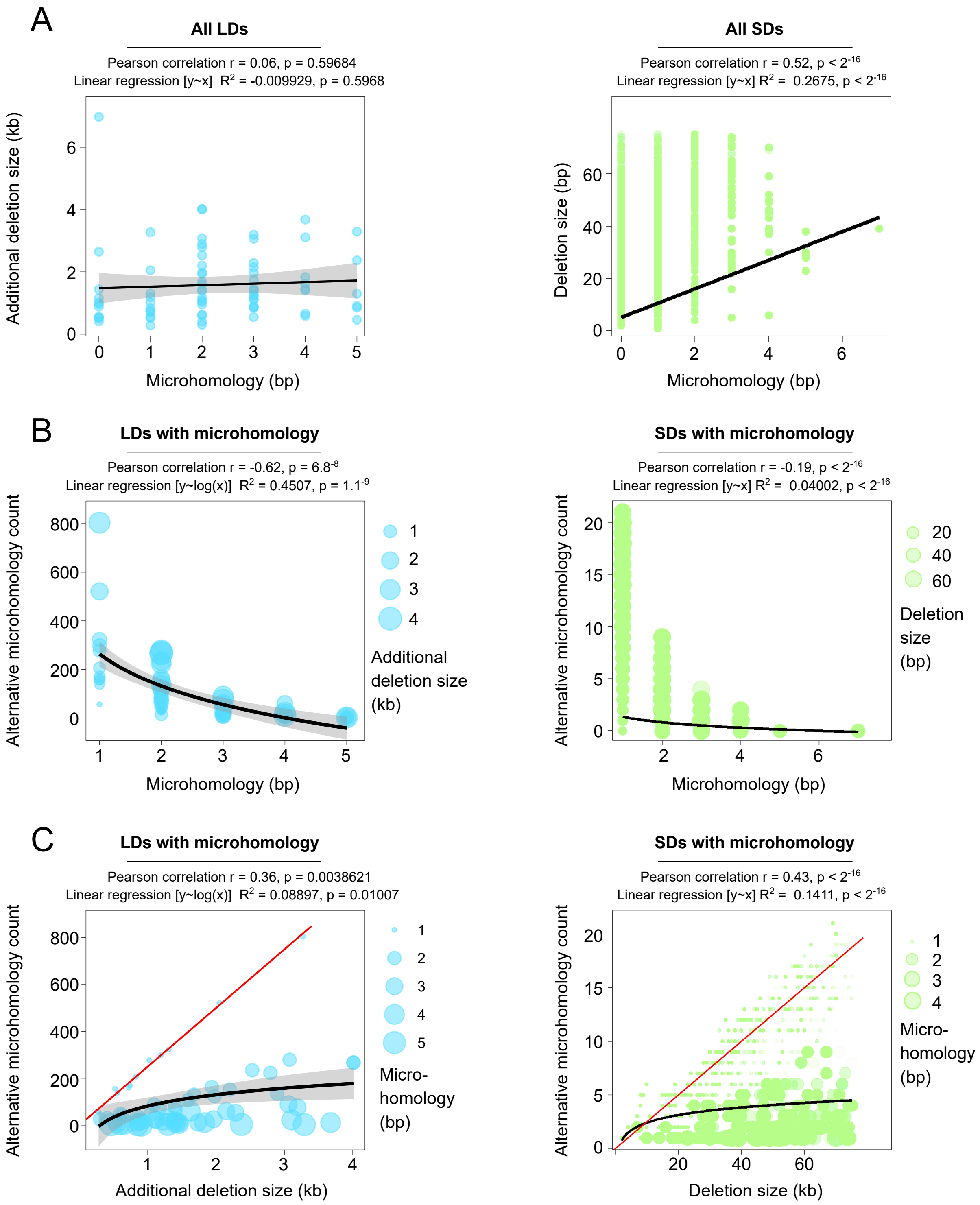

D

LDs with microhomology

Multiple linear regression
alt MH count ~ log(additional del size) + log(MH size)

Adjusted R^2 = 0.6545, p = 5.3^{-15}

log(MH size) residual = -212.69 (p = 1.8^{-14})
log(additional del size) residual = 99.17 (p = 9.0^{-8})

SDs with microhomology

Multiple linear regression
alt MH count ~ log(del size) + log(MH size)

Adjusted R^2 = 0.5091, p < 2^{-16}

log(MH size) residual = -3.065292 (p < 2^{-16})
log(Del size) residual = 1.395308 (p < 2^{-16})

Supplementary Figure 9

A

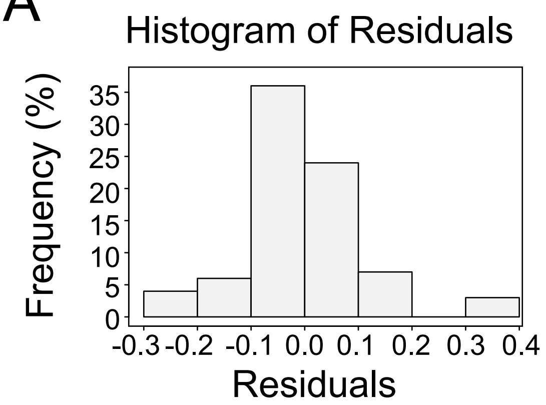

B

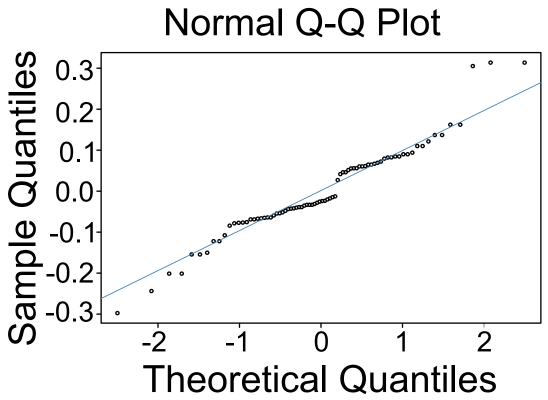

C

Multiple linear regression summary

```
Call:
lm(formula = deletionFrequency ~ log(proximity) + cutEfficiency, data = All)

Residuals:
     Min       1Q   Median       3Q      Max
-0.21981 -0.06501 -0.02923  0.06411  0.30111

Coefficients:
            Estimate Std. Error t value Pr(>|t|)
(Intercept)  0.624518   0.070606   8.845 1.57e-12 ***
log(proximity)   -0.103572   0.005979 -17.323  < 2e-16 ***
cutEfficiency       0.250865   0.083411   3.008  0.00382 **
---
Signif. codes:  0 '***' 0.001 '**' 0.01 '*' 0.05 '.' 0.1 ' ' 1

Residual standard error: 0.1067 on 61 degrees of freedom
Multiple R-squared:  0.833,  Adjusted R-squared:  0.8275
F-statistic: 152.1 on 2 and 61 DF,  p-value: < 2.2e-16
```

Supplementary Table 1 – single guide RNAs used in the study	
**sgRNA Name**	**Sequence 5' to 3'**
**Site1_sgRNA1**	TTGGTTAGTCATGTGCGTCT
**Site1_sgRNA2**	CCAAAATGTCTGCAGCCAGT
**Site1_sgRNA3**	GCCTAGTTCACTTGAGCCTT
**Site1_sgRNA4**	CGCGTGTCCTTTCCTTGGAC
**Site2_sgRNA1**	CCCTTGAATGGGATTCATGC
**Site2_sgRNA2**	AGAGAGTCCTGATCTAAATG
**Site2_sgRNA3**	GTACTTTTCAGAGAGCAAGT
**Site2_sgRNA4**	AATTATAGTACGTGTCTGTC
**Site3_sgRNA1**	AGACCAACCAGTCCTAAGAT
**Site3_sgRNA2**	ACCAGAGGATCAGCACTTGG
**Site3_sgRNA3**	GTTGCTCAGTGGCCCCTTCT
**Site3_sgRNA4**	GGCATCCCCTTTATCGCCGA
**Site4_sgRNA1**	GACTGATCCTCGCGCCGTCG
**Site4_sgRNA2**	AGCCCCGACATGACCGTGAA
**Site5_sgRNA1**	GGGTCTCCATAGGGCAAGGC
**Site5_sgRNA2**	GAGTCCTGTGATGATAGTCA
**Site6_sgRNA1**	ACAGCCGATTTTGTAGGCAA
**Site6_sgRNA2**	ACTTCAGATCAGCTTACCTT
**Site7_sgRNA1**	GTCCAACTGAAAACGCCCTT
**Site7_sgRNA2**	CTCTGTGACTAGGTCTCTGA
**Site8_sgRNA1**	CACACCGCCCCCCACTTCTT
**Site8_sgRNA2**	TACAGACCACCAGGAAAGTC
**Site9_sgRNA1_D10A**	GGTATGTAAAGCAAGGGACT
**Site9_sgRNA2_D10A**	ACTCCCTAGTAGCTAGCCGT
**Site9_sgRNA3_D10A_**	GCCCACGTGTGAATATTTTA
**Site9_sgRNA4_D10A**	TGCATATTCCTGGAAGATAG
**Site9_sgRNA1_Cas9**	GAAATATTCACACGTGGGCA
**Site9_sgRNA2_Cas9**	GTACCAACGGCTAGCTACTA
**Site10_sgRNA1**	TGATTTCCTCCACCCCTATC
**Site10_sgRNA2**	TCATCTTGGTGAGCTTGCAG
**Site10_sgRNA3**	ACCTGCGAAGGACCCTAAAT
**Site10_sgRNA4**	GGAGGGCAAGAGACCTGCGA
**Site11_sgRNA1**	AATGCGATGCTAGCCGTGGA
**Site11_sgRNA2**	ACGGCTAGCATCGCATTTAA
**Site11_sgRNA3**	CCCCTCCGTGCTCTGCGGAC
**Site11_sgRNA4**	GCCTGTCCGCAGAGCACGGA
**Site12_sgRNA1**	GCCTGCTAGCCCCCACCGCA
**Site12_sgRNA2**	GCCTTGCGGTGGGGGCTAGC
**Site12_sgRNA3**	TGATCCGGGAACATAAACGT
**Site12_sgRNA4**	CCGGAAGTAAAAGGGCTAGA
**Site13_sgRNA1**	CAAATAAGAGTGAGCACTAC
**Site13_sgRNA2**	GAGTGAGCACTACAGGTATG
**Site13_sgRNA3**	TAACTTTGAGAGCGGAAACA
**Site13_sgRNA4**	TTCAGAATTGGCAGTAATCA
**Site14_sgRNA1**	TTATGAGATAGTTAATAAAC
**Site14_sgRNA2**	AGTGATTGTGTAATGAAAAC
**Site14_sgRNA3**	GTGCCTGGTTTTATGGGCAC
**Site14_sgRNA4**	ACTCCTGTGCCCATAAAACC
**Site15_sgRNA1**	CAGCCCCTGTAGTCGTTTGA
**Site15_sgRNA2**	CTAACCTTCAAACGACTACA
**Site15_sgRNA3**	CCCAACCAGTTCCAGTTTAG
**Site15_sgRNA4**	ACACGGGGCTTCAAGATGCT
**Site16_sgRNA1**	GACAAGAAATTCCCCACCCG
**Site16_sgRNA2**	CATTCTCAGTGGCTATCTTC
**Site16_sgRNA3**	GAGCACCCGGTGTCCATGCC
**Site16_sgRNA4**	CTTAGGCTGACCTATCTGCA

Supplementary Table 2 – Primers used in the study	
**Primer Name**	**Sequence 5' to 3'**
**Site1_SR_FW**	GGGTTAGGAGTGTCCCTGATGT
**Site1_SR_RV**	GAGGCAGCTTTGGAAGGGAAAT
**Site1_MR_FW**	AGGTTGTCAGAACAAGCTACA
**Site1_MR_RV**	AGGGAGGAGTAAGGACGAATA
**Site1_LR_FW**	CTGGAATTTCGTGACACAGAG
**Site1_LR_ RV**	CCCAAGCTCCCAAACTAAAC
**Site1_5'3kb_F**	ACGCACGAGGAGAAGAACATG
**Site1_5'3kb_probe**	CAGACACTGACTGGATCTCTCTTGCA
**Site1_5'3kb_R**	TCCACACTCTCAACTCACAGAAG
**Site1_5'1kb_UPL52_F**	TGACAGTGAATTTCTCCTCACC
**Site1_5'1kb_UPL52_R**	TCCAGCCTCATATTCGTCCT
**Site1_5’500bp_F**	CCACTCTGTCTGGAGGAGAGA
**Site1_5’500bp_probe**	TGCCAACGGAGAATGAGAGGTGTTA
**Site1_5’500bp_R**	TGCAAGGGTTCAGGGATAAGTC
**Site1_5’CR-LOA_F**	GGATGCCTCCCTTCAAAGTAAC
**Site1_5’CR-LOA_probe**	CGTTGGAACAGTGACCGCAGCC
**Site1_5’CR-LOA_R**	GCCAAGCATTTGTCTCCCA
**Site1_CR-LOA_F**	GCCACCACTTTCCTACACAA
**Site1_CR-LOA_probe**	ATCCTGGGTCTTTCTTCACAAGGGTC
**Site1_CR-LOA_R**	GGAGGCTTGCACCTTCTCAT
**Site1_3’CR-LOA_F**	CCACCAAAGGCTCAAGTGAAC
**Site1_3’CR-LOA_probe**	TAGGCTGATGTCCCCGCGTGT
**Site1_3’CR-LOA_R**	CCCTCCCTGTCCAAGGAAA
**Site1_3’500bp_F**	CTGTGGCCTCAAGTGTAGTC
**Site1_3’500bp_probe**	TGCCTTAGCGAGTGATCTGTGTGC
**Site1_3’500bp_R**	GGCCGCTCCTTTCTTCTGA
**Site1_3'1kb_UPL16_F**	CCTGTTGCACCTGGTTCTTAG
**Site1_3'1kb_UPL16_R**	TGAAGTCACATAAAATGTTTCATTCC
**Site1_3’3kb_F**	GCAGGCCACTCTGGTATGAA
**Site1_3’3kb_probe**	TGGCAGCGTCATGGTGGACTCT
**Site1_3’3kb_R**	GGAGACAGTACAAAGCACCATGAC
**Site2_SR_FW**	CCTCAGAGGCCAGAAGTACCTAAT
**Site2_SR_RV**	TTGGTGGTCTTTTCCCCGTG
**Site2_MR_FW**	TGCTCAGCAGAATTGAGGTC
**Site2_MR_RV**	CTGCACAGCTCGGAGTATTT
**Site2_LR_FW**	GCAGAATCTTCGGAGCTATTC
**Site2_LR_ RV**	GTAAGCTGAGCAGTGGTATTT
**Site3_SR_FW**	TTCCTCCCATACCTCTCTCTGATATTG
**Site3_SR_RV**	GAGATGCTGGAGTTCCCTGAGATA
**Site3_MR_FW**	GCTGCTCTCAACACAGTACAA
**Site3_MR_RV**	AGATCATTTGATGAAAGGCCTCA
**Site3_LR_FW**	GAGCATCCCTCAGAAGAGAA
**Site3_LR_ RV**	CAGTGTGGTCAAGCTGATATG
**Site4_SR_FW**	CCCGCTACTGTCCACATATT
**Site4_SR_RV**	AGAGGCTTTGAGAAGAGATGAG
**Site4_MR_FW**	GAAGTGGCACCGAGTCATTTA
**Site4_MR_RV**	CCTGATCGAGCTTCGAACTAAC
**Site4_LR_FW**	TAATCTCGTGGTCTCAGAGT

**Site4_LR_RV**	CAGCAGGATGGGAATCTAAC
**Site5_SR_FW**	ACTTAAGTGTCCACTCCGATTA
**Site5_SR_RV**	GGGATTAAGCACTTCTTTAGGC
**Site5_MR_FW**	CCACCTATTGACCTCTTCGTTC
**Site5_MR_RV**	TGCTACTGACTAATTTGAGGGTATT
**Site5_LR_FW**	TAGTTGGAAGGCTGGGATAG
**Site5_LR_RV**	AAGCTGGCAAGTAATGGAAT
**Site6_SR_FW**	TCCTTTCGTCCTCTGATGTA
**Site6_SR_RV**	CACCTGAGAGAACACAATACC
**Site6_MR_FW**	TCCATTGTCCACTGCTTTAC
**Site6_MR_RV**	GTATACAATTCTGCCCTAGCC
**Site7_SR_FW**	GTGGTCACTAGCTGTCTTCC
**Site7_SR_RV**	GAAGTTGTGTAATGGCCACAG
**Site7_MR_FW**	GTATGCAGTTAGCCACAGAG
**Site7_MR_RV**	GCGGTGTGAATGATATCCAA
**Site7_NGS_FW**	ACACTCTTTCCCTACACGACGCTCTTCC
	GATCTAGCACTGAAATCTGGTGCAT
**Site7_NGS_RV**	GACTGGAGTTCAGACGTGTGCTCTTCC
	GATCTCTTGCCCTCAGAGACCTAGT
**Site8_SR_FW**	CCTTGTCAACAGCTCACTTT
**Site8_SR_RV**	CCTGCTCTAGCTTTGTTCTG
**Site8_MR_FW**	GCATATTTCCCTCTCAGAGTC
**Site8_MR_RV**	GAAATGGATAGGAGCAGGAAG
**Site9_SR_FW**	CTCCGTAGCCTCTCCTAATGAT
**Site9_SR_RV**	CTACACAGGAGTCTCTGAATCCAT
**Site9_MR_FW**	TGCTGTAGCAAGCTTAGGGTGT
**Site9_MR_RV**	GCGAAAAGACTGCCTCAAGTTC
**Site9_NGS_FW**	ACACTCTTTCCCTACACGACGCTCTTCC
	GATCTGAGGCAGAGATGACACTGAA
**Site9_NGS_RV**	GACTGGAGTTCAGACGTGTGCTCTTCC
	GATCTGCAATGAGCTTCTGGTTGAC
**Site9_5’3kb_F**	AGCACACAACAGTCCTCTCA
**Site9_5’3kb_Probe**	CGAAAGCTCTTCCTGTCATTGTCTTTATGA
**Site9_5’3kb_R**	CCACTACAAGCATGGGACAG
**Site9_5'1kb_UPL52_F**	CCTAGGCGGCTAACCTCAC
**Site9_5'1kb_UPL52_R**	GAGAAGGGTAGGCAATGGTG
**Site9_5'500bp_UPL63_F**	GACCTCATGACCAATACCATGA
**Site9_5'500bp_UPL63_R**	GTTCTGAGGGTCTTGGAGGA
**Site9_5’CR-LOA_F**	GCCGTTGGTAATAGTTGGATCATTC
**Site9_5’CR-LOA_Probe**	TCACTATTGTGAGAGCTCCCGCTG
**Site9_5’CR-LOA_R**	CGCACTGTGAGGGAAACAAAC
**Site9_CR-LOA_F**	AGTGCGGAGGGCAGATTAAGG
**Site9_CR-LOA_Probe**	CGGCGACATCGCTGTGTTTGCAT
**Site9_CR-LOA_R**	AGGGTGGATGCCAGCAGATG
**Site9_3’CR-LOA_F**	TGCAGCCTGTGATGAGGTTAG
**Site9_3’CR-LOA_Probe**	AGCCCCTGCCCACGTGTGAATAT
**Site9_3’CR-LOA_R**	CTGAAGGTGTCACCCCTATCTTC
**Site9_3'500bp_UPL16_F**	GCTCATGACCTTTAACTTCCTACAC
**Site9_3'500bp_UPL16_R**	TTTCTGAAAAGGGTAACATAAGTGAGT
**Site9_3'1kb_UPL38_F**	CGGGCTGAGCTTATAAATCG

**Site9_3'1kb_UPL38_R**	CCTGGTGGATCTGACAGACA
**Site9_3'3kb_UPL34_F**	TGCATAGATGCGATGTTGTTT
**Site9_3'3kb_UPL34_R**	CCACCGTGGAGGAAGTTACA
**Site10_SR_FW**	TTCTGATGAGTGTTGGCGGA
**Site10_SR_RV**	CTAGGTAGAGTTCGCGGGAG
**Site10_MR_FW**	CAGGTCCAGAGCTGAGTCTTTC
**Site10_MR_RV**	CCCATATCAGAAGTGAGCCTCT
**Site10_5'3kb_UPL60_F**	CACACTTACGGCAACTTTATCCT
**Site10_5'3kb_UPL60_R**	TGGCTCCAAAACATGAAGC
**Site10_5'1kb_UPL88_F**	CAGGTCCAGAGCTGAGTCTTTC
**Site10_5'1kb_UPL88_R**	GTTGCTGGGTGAGTCAGGAG
**Site10_CR-LOA_UPL59_F**	CAGAGGCAGGGGTAGGATTT
**Site10_CR-LOA_UPL59_R**	GGTCCTTCGCAGGTCTCTT
**Site10_3'1kb_UPL81_F**	GCTGACCACGCCCACTAC
**Site10_3'1kb_UPL81_R**	CCGCTTCTTAGCCAGCAG
**Site10_3'3kb_UPL69_F**	GTTTCCACATCATCGTCCATT
**Site10_3'3kb_UPL69_R**	CTTCCTGCCCTGTTTGTGTT
**Site11_SR_FW**	CCGGAGAACATGTGTAGAAGTC
**Site11_SR_RV**	ATTAGGACATGCATATGAAAACTGT
**Site11_MR_FW**	GCTAAATGGGATTAAATGTTTAACGA
**Site11_MR_RV**	GGAAGTCTGTCATACAGATCTCAATTT
**Site11_5'10kb_UPL21_F**	TGGATGACTGCCCTCAAACT
**Site11_5'10kb_UPL21_R**	GGCATGATCAGATGGTGTGT
**Site11_5'6.5kb_UPL8_F**	AGGCGGAGGACCACATTT
**Site11_5'6.5kb_UPL8_R**	ACCCTTTCTCCAAAGGTCTGT
**Site11_5'5kb_UPL12_F**	GAGAAACAAATGGGATTACTTCAGA
**Site11_5'5kb_UPL12_R**	AGAGTCCAAGCAACCACAGG
**Site11_5'3kb_UPL13_F**	TCCTCACTTTGATCCTATTTGTCA
**Site11_5'3kb_UPL13_R**	CATGGGGAGGAAGTCAATGT
**Site11_5'1kb_UPL17_F**	CATGATGGCAGGGAAGTCA
**Site11_5'1kb_UPL17_R**	TGAACAAAGAATCGGTGCAG
**Site11_CR-LOA_UPL8_F**	GCCAACCATCACAAAGGTAAGT
**Site11_CR-LOA_UPL8_R**	CAGACCAGTGAAGACAGAATGTG
**Site11_3'1kb_UPL15_F**	TCCAAGAGGTGTAAACTGTCTGAG
**Site11_3'1kb_UPL15_R**	GGACCCTTGGAGTAACTGTCA
**Site11_3'3kb_UPL11_F**	AAGGAGTGTGCTAATGGCATGT
**Site11_3'3kb_UPL11_R**	AAGACCTTCTAGGCCTTTGTGTT
**Site12_SR_FW**	ACAATGTCAACGGGGTCAGG
**Site12_SR_RV**	TGTTCGACGTGCTCACCTTC
**Site12_MR_FW**	GAGAGGGCAGGAACTTTGC
**Site12_MR_RV**	GCAAGCAAGGTGCTAACAGA
**Site12_5'1kb_UPL84_F**	GAGAGGGCAGGAACTTTGC
**Site12_5'1kb_UPL84_R**	CAAAATCTTGCCCAACAACTAA
**Site12_CR-LOA_UPL7_F**	GGCAAGAAGTTGGCAAGG
**Site12_CR-LOA_UPL7_R**	CCACATGCAGAGTGGTAAGG
**Site12_3'1kb_UPL59_F**	TACCAGGCGGTAGGTGACTT
**Site12_3'1kb_UPL59_R**	GCGAAAACAACCCTCCAG
**Site12_3'3kb_UPL3_F**	ATCGGACTGTCTTGCTCTGG
**Site12_3'3kb_UPL3_R**	CTTTTCCCCTTGCAGCTATTT
**Site13_SR_FW**	CGGCGTTAGCTGGACTTCAA

**Site13_SR_RV**	TGGACGAGGCTTTAGATGTGG
**Site13_MR_FW**	CGACTCCACCCCATTTCTT
**Site13_MR_RV**	GCTGCACCTACATTGAGATCC
**Site13_5'3kb_UPL50_F**	AGGTTCATTCCCTAGAGGCTTT
**Site13_5'3kb_UPL50_R**	AAGAACTAACAGCCCCAATCC
**Site13_5'1kb_UPL10_F**	CGACTCCACCCCATTTCTT
**Site13_5'1kb_UPL10_R**	CGACAGCCCCATACTGAGAC
**Site13_CR-LOA_UPL4_F**	CCCTGCAGGTTACTGGAGAA
**Site13_CR-LOA_UPL4_R**	AGTGTGCGTGGTCACTGG
**Site13_3'1kb_UPL24_F**	CTGGGGAGCAGCTCTTGT
**Site13_3'1kb_UPL24_R**	GCTGCACCTACATTGAGATCC
**Site13_3'3kb_UPL16_F**	CAGAAGGCGGGGTCATAGT
**Site13_3'3kb_UPL16_R**	AGACCCAACCTGATTCATGC
**Site14_SR_FW**	ATTAGATGACTTTGCATCTCCCCC
**Site14_SR_RV**	TGTCATCCTACTTGGCCCTCT
**Site14_5'1kb_UPL38_F**	GCTGCATAATGAGAGCAGTGA
**Site14_5'1kb_UPL38_R**	TCGAGGCTGATGTGAGTGAC
**Site14_CR-LOA_UPL7_F**	CCGACATCCAATACAAGCAAT
**Site14_CR-LOA_UPL7_R**	GGTTTTGCTGTCATCTGTGG
**Site14_3'1kb_UPL26_F**	TGACTTGCTCATCCTATCTTGC
**Site14_3'1kb_UPL26_R**	GAGGCCCCTGTAAGTGGTG
**Site15_SR_FW**	ACACCTGGTGACCTTTTCCC
**Site15_SR_RV**	AGACTCAAATGCCTCTGGGC
**Site15_5'1kb_UPL3_F**	AGGAGGAAAGGCAAAGAGGA
**Site15_5'1kb_UPL3_R**	GCAAAGCCCAGATCATGC
**Site15_CR-LOA_UPL57_F**	CACAGACATTCATGGTATTGAACA
**Site15_CR-LOA_UPL57_R**	CATGAAGCTTCTGATGGGATTA
**Site15_3'1kb_UPL21_F**	CCCCCAATGGTCTTTTTACTC
**Site15_3'1kb_UPL21_R**	GGGACTTTATGAGCCCAAGAC
**Site16_SR_FW**	AGGTGGTCCATTCATGCTGC
**Site16_SR_RV**	GTGAAGGGAGAAATGATCGCC
**Site16_5'3kb_UPL4_F**	TCGCCCCTTCTTTTCAGAC
**Site16_5'3kb_UPL4_R**	TGTATGCCTTTAGTCCCAGCA
**Site16_5'1kb_UPL10_F**	TGATGACATCTTTGATCCCAGT
**Site16_5'1kb_UPL10_R**	GCTGGCCTCGAACTCAGA
**Site16_CR-LOA_UPL21_F**	ACTCTCGGACGCAGGGTAA
**Site16_CR-LOA_UPL21_R**	GTTCCATCTCTCCTGGCTCA
**Site16_3'1kb_UPL15_F**	TCCGACGAAGCAATAGCAGc
**Site16_3'1kb_UPL15_R**	TCACTGCTGAAAGAGCACAGA

Supplementary Table 3 - Summary of gene editing experiments in mouse zygotes in vivo

		By PCR		By ddPCR				
**Gene**	**MGI ID**	**No. of animals assessed**	**No. of animals with a deletion**	**No. of animals assessed**	**No. of animals with a deletion**	**No. of animals with a larger than expected deletion**	**No. of control littermate animals assessed**	**No. of animals with a larger than expected deletion**
Ajuba	1341886	21	9	11	9	0	2	0
Ap3b2	1100869	12	9	10	10	**1**	1	**1**
Arhgap42	1918794	11	5	7	5	0	2	0
Cckbr	99479	16	9	11	11	**2**	2	**2**
Col1a2	88468	21	10	13	9	0	3	0
Fam19a2	2143691	13	5	8	8	**3**	3	**2**
Gch1	95675	13	9	12	10	**2**	3	**2**
Gls	95752	20	1	2	1	0	1	0
Gria1	95808	7	7	7	7	0	0	0
Hpca	1336200	19	6	8	6	0	2	0
Itga11	2442114	11	5	9	5	0	4	0
Itsn1	1338069	13	2	3	2	0	1	0
Kcnma1	99923	18	6	8	6	0	2	0
Limd1	1352502	6	2	4	2	0	2	0
Nedd1	97293	20	6	8	5	0	2	0
P3h2	2146663	12	4	8	4	0	4	0
Pcdh8	1306800	10	4	10	8	**4**	6	**4**
Pde2a	2446107	8	4	8	4	0	4	0
Prkra	1344375	15	6	8	6	0	2	0
Rgs2	1098271	6	3	6	6	0	3	0
Rnf168	1917488	23	10	17	10	0	7	0
Rsad2	1929628	7	1	3	1	0	2	0
Sec31a	1916412	57	5	8	5	0	3	0
Senp5	2443596	10	4	5	5	**1**	1	**1**
Shisa9	1919805	6	4	6	4	0	2	0
Slc17a7	1920211	8	3	8	4	**1**	5	**1**
Slc9a7	2444530	4	2	4	2	0	2	0
Tbx15	1277234	6	2	3	2	0	1	0
Tfrc	98822	11	9	11	9	0	2	0
Th	98735	26	3	4	2	0	1	0
Tpo	98813	6	3	5	2	0	2	0
Trpc1	109528	18	5	7	5	0	2	0
Usp31	1923429	14	4	5	3	0	1	0

Supplementary Table 4 - Summary of all deletions analysed per site					
**Site number**	**Chromosome**	**Gene locus**	**Description**	**Larger Deletions**	**Expected Deletions**
1	16	*Runx1*	Non-coding	12	5
2	16	*Runx1*	Non-coding	4	5
3	16	*Runx1*	Non-coding	3	5
4	16	*Runx1*	Non-coding	8	14
5	16	*Runx1*	Non-coding	12	13
6	16	*Runx1*	Non-coding	1	3
7	16	*Runx1*	Non-coding	11	3
8	16	*Runx1*	Non-coding	3	2
9	6	*Prickle2*	Exon 6	13	2
10	7	*Cckbr*	Exon 2	1	1
11	10	*Fam19a2*	Exon 3	1	1
12	14	*Pcdh8*	Exon 2	1	1
13	7	*Slc17a7*	Exon 2	1	1
14	4	*Elavl4*	Exon 2	1	1
15	9	*Scn11a*	Exon 3	1	1
16	10	*Trpm2*	Exon 3	1	1
**Totals**	**7**	**9**		**74**	**59**

Supplementary Table 5 - All mapped deletion sequences
Larger Deletions (LDs)

ID	Site	Upstream (retained)	Upstream (lost)	Downstream (lost)	Downstream (retained)	MH score	MH	Notes
LD1	1	CGTGGCGAGG	GCTGAGCTAT	TGTTAGCACA	GCTTCTCCAC	3	GCT	
LD2	1	ACGTTATGAT	AGTACGGAAA	AGTCAGGCTC	AGCCACCATG	2	AG	
LD3	1	CAAAATGTCC	TGCAGCCAGT	TGTTCTGTAG	CGTCAAACTG	0	No_MH	Ins CC
LD4	1	GAACCCTTGC	ACAGGGAGGA	AAAACTTGAT	ACAGCAACTC	4	ACAG	
LD5	1	GTCCAAGGAA	AGGACACGCG	ATTTAGGCAG	AGTCCTCCCT	2	AG	
LD6	1	AGCCGAGACG	CACATGACTA	GTTCCTGGGC	CACAAACATG	4	CACA	
LD7	1	GGACGAATAT	GAGGCTGGAG	TGTACATATG	GAGGTCAAAG	4	GAGG	
LD8	1	CTTTCTCAGG	TGGCCTGTCA	ATGAGCCATT	TGTATGCGTC	2	TG	
LD9	1	GCCCTTTCCA	GACCTGTCTC	CTTTGTTCAC	GGGACACCTG	1	G	
LD10	1	ATCCAACTGT	ATCTATAAAG	CGCCTGCTGA	ATCATCCTCT	3	ATC	
LD11	1	GGTTCTGAAA	ACATATTTGA	CCGACTCCTC	ACCGCACTGC	2	AC	
LD12	1	AGCTCTAATG	AGGAATAGCA	AGTTAAGTTT	TATTTGTAGC	0	No_MH	
LD13	2	TGCAGGAGTA	CCCTGAGATG	GCACAGAGGC	CCCTGCGATG	5	CCCTG	
LD14	2	GTGTACTTTT	CAGAGAGCAA	GTCCCTGCCC	CAGAGTCCCC	5	CAGAG	
LD15	2	CAACAACGTT	AGCCACTCAG	CCACCACCGA	AGTCAGAGAG	2	AG	
LD16	2	AGTCAGTCAG	GGTGTAGTGT	GCAGACCACT	GGTGTCTGGT	5	GGTGT	C to t mut
LD17	3	TGTTAGCTTA	CTTAGCAGTC	ACTCTGGGGT	CTGCTCATCT	2	CT	C to t mut
LD18	3	GGGGGTCCAC	GCTGCAGAGT	CTCACAAGGT	GCACAGACAC	2	GC	
LD19	3	CAGAAGGCCC	TCGGCGATAA	ACACATATAT	ACACAGAAAC	0	No_MH	
LD20	4	GGCCTTTCAC	GGTCATGTCG	TTCCCGAGTG	GGCCCGACTT	2	GG	
LD21	4	GGGCATTTTC	GCCGAAGAGC	GGTCTCTACT	GTCGTGATCC	1	G	
LD22	4	CCCGGCGGTG	GCTGGTTTTC	ACTTTCTCTC	GCTTGCCTCA	3	GCT	
LD23	4	AGCCTGGAAG	TGAAAGTTGT	AGCCCCGACA	TGACCGTGAA	3	TGA	
LD24	4	CAACTGCAAG	GAGTTGGTCT	GTAAAGCACT	GACCATTTAT	2	GA	
LD25	4	AATTTTATGA	GGACAAAATG	GACCATTCCC	GGATGCCAGC	3	GGA	
LD26	4	AAATAAATGG	TCAGTGCTTT	TTAGACCAAC	TCCTTGCAGT	2	TC	
LD27	4	CAGGCAGACT	CGCACTCTTC	GCCCCGACGG	CGCGAGGATC	3	CGC	
LD28	5	TAGGGCAGGG	AGGCGGGCAG	GTTTTTCATT	GTATGGTTAT	0	No_MH	Ins GGG
LD29	5	GGTCTCCATA	GGGCAAGGCG	TCATTGTATG	GTTATTCTGT	1	G	
LD30	5	CGTCTTTTAT	GAAGCTTATT	GATAGTCATG	GAAGTCCAAG	4	GAAG	
LD31	5	GGCCTTCCTT	TCCTTACTTG	AGCCTTCTGG	TCCACTTCTG	3	TCC	
LD32	5	TTCCTGGGAT	CCCCAGGCAG	GCGGGCAGAT	GAAAGCTTTC	3	GAT	
LD33	5	TCCCAAAATC	GAGACACTTA	CAGATTCTCT	GGACAGAAAG	1	G	
LD34	5	CTGTGGGTGT	GAGCTCTTCT	AATTAATGTC	GAGAGTGCTG	3	GAG	
LD35	5	CTTCAGCCTT	CCAGAAGTCA	GTTAAAGCCG	CTCTCTAAGG	1	C	Ins T
LD36	5	TGGTGAGTCC	TGTGATGATA	CACACACACG	TGTGTGTATG	4	TGTG	
LD37	5	GGTCTCCATA	GGGCAAGGCG	GCAGGGGTAT	GGATGAATAC	2	GG	
LD38	5	GGCTTACACT	TCTGACACTG	GTGATGATAG	TCATGGAAGT	2	TC	
LD39	5	GAAGTTTTGT	GGAGAGCAAA	ATCAGTCGCA	GTCCCTGGCC	1	G	
LD40	6	CACCTTCCCC	AGCCCCAAGT	GCCCAAGGTA	AGCTGATCTG	3	AGC	
LD41	7	GGTGGTCACT	AGCTGTCTTC	CCGCAAACAA	AGGAAGCTTT	2	AG	
LD42	7	AGGGGTTAAA	ACCTAAGGGC	CCAGGATATC	ACCTCCATCT	4	ACCT	
LD43	7	AGCGAGCAAG	GGGATTCCGA	ACCTAAGGGC	GTTTTCAGTT	1	G	
LD44	7	AAAGAACTTC	ATACACTGGC	ATCATCCAGG	ATATCACCTC	3	ATA	
LD45	7	AATCATGTGG	GTTCCCACAG	AATTCCAGGA	GTGTTCATTA	2	GT	
LD46	7	TCACTCTGCC	CAGCACACCG	CCTCCCCCTT	CAGCGTTAGA	4	CAGC	
LD47	7	AAGGGGGAGG	TCGTGGGTCC	ATCCCCTTGC	TCGCTCGTTC	3	TCG	
LD48	7	CTTCTTGCTG	GTCTCAAGTT	CACTGGCGTT	GAAGCTGCAG	1	G	
LD49	7	CAACTGAAAA	CGCCCTTAGG	TCATGATACT	CGGGTTGATT	2	CG	
LD50	7	AAAACGCTCT	TAGGTTTTAA	AGGCCCGGTA	GCTACTTATT	0	No_MH	C to t mut
LD51	7	CTAATCATGT	GGGTTCCCAC	ACCTGCTGGG	GGGCAGAGAT	3	GGG	
LD52	8	TTCCTAAAGA	AACCAGACTT	AGTGTGTTGG	AACACCCAGG	3	AAC	
LD53	8	CAGAGAGGAG	GGAGGGGGGA	CTCTGTGAAA	AGGTCAATAG	0	No_MH	Ins AG
LD54	8	CAGCACCAAG	CATTTGAAAG	CCAGACTTTC	CTGGTGGTCT	1	C	
LD55	9	GGGTCATTAT	CCTTGAAATG	ACTCTCCAGC	CCTTGCTAGA	5	CCTTG	
LD56	9	GGAGAGTTGA	GCACTTCAAC	AGATGCTCTC	GAGTTGTTCA	1	G	Ins GAGT
LD57	9	GAACAGAGAG	CATCTTTATA	TCAGATCCAC	CAGGACAAGG	2	CA	
LD58	9	TATTTAAGAG	TAAATGGGTT	AGTCAATGGA	TACCTATCTG	2	TA	
LD59	9	TCTGTGAATT	GGAGTCCCCT	AGGAGAAGGA	GGAGGAGTGG	4	GGAG	
LD60	9	ATCCAGATAG	GTATCCATTG	GACCAGATTT	GTTCAGTTAT	2	GT	
LD61	9	GAGCACTTCA	ACCTTACCAT	ATTCACACGT	GAGCAGGGGC	0	No_MH	Ins GA
LD62	9	TTTTTTTTCC	TAGAGAATCT	TTAACATCAT	TAGGAGAGGC	3	TAG	

LD63	9	AACTGAACAA	ATCTGGTCAT	AACATCACCC	ATGCCCCAGT	2	AT	
LD64	9	CGTATTGTGT	ATATGTCTAT	ACATAAACCC	ATTTACTCTT	2	AT	
LD65	9	CAAGCTTTCT	GCTCTGGTGT	GTGGGCAGGG	GCTCTAACCT	5	GCTCT	
LD66	9	TGCTTCTAGT	AGGGAAAAAT	AGGAGGGGTG	AGGTTAGCCG	3	AGG	
LD67	9	AGGGCTGGAA	GAGTTGAGCA	CCTTTCCTCT	ACCACACCCT	0	No_MH	Ins A
LD68	10	TTTTAGTTTT	AGAGAACATA	GGGGAGGGCA	AGAGACCTGC	5	AGAGA	
LD69	11	GGGCTCAAGA	CCGAGGTCCA	ATGCAAAGTT	CCTGCCCTCT	2	CC	
LD70	12	CAGGTCAGGA	CTCTCCCGTC	ACGTTTATGT	TCCCGGATCA	0	No_MH	Ins A
LD71	13	ATGGAAACAC	CATGTTTCCG	GAAAGTACAT	ATGCTTTCAT	0	No_MH	
LD72	14	AAGAATGTGA	CTAAGTAAAG	AACAAGATGA	AAGGCAAGAT	3	TGA	Ins A
LD73	15	AAGACAGACG	GACACTCTGG	GTTCCAGTTT	GGAAAACAGT	1	G	Ins GGAAAAC
LD74	16	AGTGGCTATC	TTCAGGCTGT	GGATATGAGA	ACTATGAGTT	0	No_MH	Ins AC

Expected size deletion sequences (EDs)

ID	Site	Upstream (retained)	Upstream (lost)	Downstream (lost)	Downstream (retained)	MH score	MH	Notes
ED1	1	GCCGAGACGC	ACATGACTAA	CTTTCCTTGG	ACAGGGAGGG	3	ACA	
ED2	1	CCGCAGCCGA	GACGCACATG	CCTTTCCTTG	GACAGGGAGG	3	GAC	
ED3	1	GACCGCAGCC	GAGACGCACA	CTTGGACAGG	GAGGGAGGGC	3	GAG	
ED4	1	AGACGCACAT	GACTAACCAA	CCTTTCCTTG	GACAGGGAGG	3	GAC	
ED5	1	GACCGCAGCC	GAGACGCACA	GTCCCCGCGT	GTCCTTTCCT	1	G	
ED6	2	TACAAGTAAC	ACAAGCCAGC	AGCAGCATCT	ACCACAGAAA	2	AC	
ED7	2	TGATACTTGG	TTTCAGAGAG	AGTACACAAA	TTATAGTACG	2	TT	
ED8	3	AAGTAACACA	AGCCAGCATG	AATGCTTAGC	AGCATCTACC	3	AGC	
ED9	3	TGTTAAGAAG	ATACAAGTAA	AAGTACACAA	ATTATAGTAC	2	AT	
ED10	3	AACACAAGCC	AGCATGAATC	GAAAATGCTT	AGCAGCATCT	4	AGCA	
ED11	3	AGCCCTAGCA	CTTCCCATCT	CGCCGAGGGC	CTTCTGGATC	4	CTTC	
ED12	3	CTAGCACTTC	CCATCTTAGG	TCGCCGAGGG	CCTTCTGGAT	2	CC	
ED13	3	ACAGCCCTAG	CACTTCCCAT	GAGGGCCTTC	TGGATCCTGA	0	No_MH	
ED14	3	CACTTCCCAT	CTTAGGACTG	ATGGCATCCC	CTTTATCGCC	3	CTT	
ED15	3	TTAGGACTGG	TTGGTCTGTG	GCATCCCCTT	TATCGCCGAG	1	T	
ED16	4	CATAGGGCAA	GGCGGGCAGA	GTGATGATAG	TCATGGAAGT	0	No_MH	
ED17	4	GGGGTCTCCA	TAGGGCAAGG	GTGATGATAG	TCATGGAAGT	1	T	
ED18	4	TCCATAGGGC	AAGGCGGGCA	TGTGATGATA	GTCATGGAAG	0	No_MH	
ED19	4	TCCATAGGGC	AAGGCGGGCA	GTCATGGAAG	TTCCAAGCAA	0	No_MH	
ED20	4	TGGGGGTCTC	CATAGGGCAA	TGATGATAGT	CATGGAAGTC	3	CAT	
ED21	4	CATAGGGCAA	GGCGGGCAGA	GTGATGATAG	CTCATGGAAG	0	No_MH	Ins C
ED22	4	CCATAGGGCA	AGGCGGGCAG	GTGATGATAG	TCATGGAAGT	0	No_MH	
ED23	4	CATAGGGCAA	GGCGGGCAGA	CTGTGATGAT	AGTCATGGAA	0	No_MH	
ED24	4	CTCCATAGGG	CAAGGCGGGC	TGATGATAGT	CATGGAAGTC	2	CA	
ED25	4	CCATAGGGCA	AGGCGGGCAG	TGATGATAGT	CATGGAAGTC	0	No_MH	
ED26	4	CATAGGGCAA	GGCGGGCAGA	GTGATGATAG	TCATGGAAAT	0	No_MH	
ED27	4	GGGGGTCTCC	ATAGGGCAAG	GTGATGATAG	TCATGGAAAT	0	No_MH	
ED28	4	CATAGGGCAA	GGCGGGCAGA	TGATGATAGT	CATGGAAGTC	0	No_MH	
ED29	5	AGGCCTTTCA	CGGTCATGTC	CGCCCCGACG	GCGCGAGGAT	0	No_MH	
ED30	5	CAGGCCTTTC	ACGGTCATGT	CACGCCCCGA	GCGGCGCGAG	0	No_MH	Ins G
ED31	5	GCAGGCCTTT	CACGGTCATG	CACGCCCCGA	CGGCGCGAGG	1	C	
ED32	5	GGCAGGCCTT	TCACGGTCAT	CGACGGCGCG	AGGATCAGTC	0	No_MH	
ED33	5	CAGGCCTgTC	ACGGTCATGT	CACGCCCCGA	CGGCGCGAGG	0	No_MH	T to g mut
ED34	5	TTCACGGTCA	TGTCGGGGCT	GATCAGTCTC	ACCTGGAGGT	0	No_MH	
ED35	5	TTCACGGTCA	TGTCGGGGCT	CACCTGGAGG	TCCTTAGGGT	1	T	
ED36	5	AGGCCTTTCA	CGGTCATGTC	GCCCCGACGG	CGCGAGGATC	2	CG	
ED37	5	AGGCCTTTCA	CGGTCATGTC	CACGCCCCGA	CGGCGCGAGG	3	CGG	
ED38	5	GCGGCATGGA	GGATCAGGCA	CCGACGGCGC	GAGGATCAGT	1	G	
ED39	5	AGGCCTTTCA	CGGTCATGTC	ACGCCCCGAC	GGCGCGAGGA	0	No_MH	
ED40	5	CAGGCCTTTC	ACGGTCATGT	GCACGCCCCG	ACGGCGCGAG	4	ACGG	
ED41	5	CAGGCCTTTC	ACGGTCATGT	CACGCCCCGA	CGGCGCGAGG	0	No_MH	
ED42	5	GCCTTTCACG	GTCATGTCGG	CGCCCCGACG	GCGCGAGGAT	3	ACG	
ED43	6	CAGACACTCC	TTTGCCTACA	CTAGCCCAAG	GTAAGCTGAT	0	No_MH	
ED44	6	CACTCCTTTG	CCTACAAAAT	CTAGCCCAAG	GTAAGCTGAT	1	G	
ED45	6	ACACTCCTTT	GCCTACAAAA	CCTAGCCCAA	GGTAAGCTGA	1	G	
ED46	7	TTAAAACCTA	AGGGCGTTTT	ACTAGGTCTC	TGAGGGCAAG	0	No_MH	
ED47	7	GTTAAAACCT	AAGGGCGTTT	TAGGTCTCTG	AGGGCAAGCT	1	A	
ED48	7	TAAAACCTAA	GGGCGTTTTC	GACTAGGTCT	CTGAGGGCAA	0	No_MH	
ED49	8	ACCGCCCCCC	ACTTCTTTGG	AAGAAACCAG	ACTTTCCTGG	4	ACTT	
ED50	8	CCGCCCCCCA	CTTCTTTGGC	GAAACCAGAC	TTTCCTGGTG	0	No_MH	

ED51	9	AACTCCCTAG	TAGCTAGCCG	GAGCCCCTGC	CCACGTGTGA	0	No_MH	
ED52	9	TGTCCATATA	ACTCCCTAGT	GCCCCTGCCC	ACGTGTGAAT	2	AC	
ED53	10	GACAAATACA	TGGGGTTTTT	GACCCTAAAT	TGGAACTCCA	3	TGG	
ED54	11	ATAGAATACC	CTCCTTCCAC	CCCAGCCTGT	CCGCAGAGCA	1	C	
ED55	12	CAGTCTTGGA	GCCTTGCGGT	CCACGTTTAT	GTTCCCGGAT	1	G	
ED56	13	TTATGAATTC	AAAACCAGTC	TGAGAGCGGA	AACATGGTGT	2	AA	
ED57	14	TATGAGATAG	TTAATAAACA	GTGCCTGGTT	TTATGGGCAC	3	TTA	
ED58	15	CCTCTCACCA	GCCCCTGTAG	GTCTGGCACT	GTGTGGGACT	1	G	
ED59	16	CTCAGTGGCT	ATCTTCAGGC	GCCTTGCAGA	TAGGTCAGCC	0	No_MH	

Simulated larger deletion sequences

ID	Site	Upstream (retained)	Upstream (lost)	Downstream (lost)	Downstream (retained)	MH score	MH	Notes
Sim1	n/a	AATAGATCTT	GCTGCATAAC	AGAGGAGGTA	CAGAGACAGT	0	No_MH	
Sim2	n/a	AAAGAGTTTA	TCTACAAAAT	AATTAAACAT	CCTCTACCTT	0	No_MH	
Sim3	n/a	ATGACCTGGT	GTTGCTGGAA	TGATTACTGT	AGCAGCTATG	2	GT	
Sim4	n/a	CTGTGTCTGC	AACCTATGCA	AGCACTGCTC	TAGATTGGAC	1	C	
Sim5	n/a	AACTCTATCA	ATAAGCTATA	AGTGACCCTG	AGTTGTGTAA	1	A	
Sim6	n/a	GGGACTTACA	TATGGATATG	TGGCTTGAGA	CCTTCAGTAT	1	A	
Sim7	n/a	ACTAGGTTTT	CATTCTCCAT	GCAGTGACTT	AGATAGTTTA	2	TT	
Sim8	n/a	CCCATCCCTT	TTTAAGGAGA	TAAAATCGAC	TTCCAACCCA	2	TT	
Sim9	n/a	AATAGAGATA	GATGCATCAA	AGAGTCGTTT	CTTAAGGCTT	0	No_MH	
Sim10	n/a	ACCTTTGTGT	AGTACCAAGG	GACTTTTTTT	CATGAAAATA	1	T	
Sim11	n/a	TCCTCCTTCT	TTTTCTTCTT	ATATATATGT	ACACTCTGTG	1	T	
Sim12	n/a	GAGATTTCAC	CCCAGCAAGA	ACAGGGCCTG	CAGAAGGGTG	1	C	
Sim13	n/a	ATTTGTATTT	TATTAAGCAT	ATATCATACA	CGTGAAAAAC	0	No_MH	
Sim14	n/a	TGGAAGCACA	AGCCTCTCTC	ACTACTTCGC	TCCATTTATG	0	No_MH	
Sim15	n/a	CTGTACAGAT	GGGAAAATGA	CAGTGGGGAG	TTCTGCATTT	0	No_MH	
Sim16	n/a	ACTAGACCAG	ACCTTTTACA	ATGGCAGCAG	GTATCCTGGG	3	CAG	
Sim17	n/a	AAAATTCAAC	AATCCTTCAT	AACAAAATAA	TTAAGGGAAG	0	No_MH	
Sim18	n/a	AAGCCTGAGG	AACAAAAGGA	GCATGGCAGT	AGGCAGACAG	1	A	
Sim19	n/a	TGCTCTGATC	ACAGATGAGA	TGCTGAAGTT	CACATCATTT	0	No_MH	
Sim20	n/a	AATTGAAAGC	CCTGACTTCG	CCTCCAGAGT	ATTAGGATTA	0	No_MH	
Sim21	n/a	GTGCTAATAC	AGTAATAAAA	GGTGAGTTCC	AGCATCCACT	2	AG	
Sim22	n/a	TAATTGAATA	TTCCCTATGG	AAATAATGTT	TGGTTGAAAT	1	T	
Sim23	n/a	AGAAAACACT	TCTGGAAATC	AAATCCATAT	GCAGAAGAAG	1	T	
Sim24	n/a	GCATGCAGCA	CTCATGAGCT	TTCTGAGATA	GCGGGCAAGT	1	A	
Sim25	n/a	ATTCAGTTGC	CTCTCCTCAC	ACGGAGGGTG	ACCTTGAACT	0	No_MH	
Sim26	n/a	TGTGTTTGTT	ATACTTGTAG	CTTTCTCATT	CCATCCTTTG	2	TT	
Sim27	n/a	TCGATTCTAG	TTCATTCTAC	AAAAAAGGTT	TATTTTGTTC	1	T	
Sim28	n/a	CACTAGTGAA	AACCAGAGTG	TCCAGTAGAA	AGACAGGGCA	3	GAA	
Sim29	n/a	AACCAATTTA	TAACTTCCCA	GAGACCCCTG	GCTCCAGCTG	0	No_MH	
Sim30	n/a	AAATTACGAG	GGAATTGGAA	AATTAATATA	AATAGCTTCT	0	No_MH	
Sim31	n/a	CAGATAAGTT	CTAGCAGAAG	GAGGAAACCA	CAGAGCATGC	1	C	
Sim32	n/a	CTGGGGTCCG	AGGCGTGGGG	TTCGAGGCAG	GGTTTCTCTG	1	G	
Sim33	n/a	TTCTTTCATT	GGGTGATATT	TGTACTCAAG	TATGTAGCCA	0	No_MH	
Sim34	n/a	GGAGTATACT	TTGGAAAAGG	TCCTCCCAGA	CACAGCTTAA	0	No_MH	
Sim35	n/a	TAAGAGAAAG	TTGTCTCAAA	GATTTGAATT	TCTCTGATCA	1	T	
Sim36	n/a	ATGGACTGGT	GACAAGTGAT	TACCCTCCCA	AGACATTTCC	0	No_MH	
Sim37	n/a	ATACAGCCTC	TTGTTTTGTA	TAAAGTAGTG	CTGGGCTGGT	0	No_MH	
Sim38	n/a	CGCAGTCTCG	AGACCTGCGG	CCACAAGTCC	TGCCGGGAAG	0	No_MH	
Sim39	n/a	CTCTTCCCAG	TGATGGCCGA	CCCTAGCACA	ACCTGCCCTG	0	No_MH	
Sim40	n/a	TTCTGATTAC	AGTCCATCAC	GTCATCGTTT	CCTTGATCTG	0	No_MH	
Sim41	n/a	ATGAAGGGAG	AAGAAGATGG	CTGCAGGACG	TTGTAATTTG	1	G	
Sim42	n/a	TACTATTGCA	AGTGCCATCC	AATGGTTGAT	CCTAGTGAGG	0	No_MH	
Sim43	n/a	TTAAACTAAA	TCAAGGGGTG	AATATAAAAA	CTTCATAATG	3	AAA	
Sim44	n/a	AATTAAGCTT	GGTCCTGCAT	ACTGTGTAAA	ACACTACACA	0	No_MH	
Sim45	n/a	TTATGGGATC	AAGACTGATC	TGGGAACAGA	ATAATTGCTG	1	A	
Sim46	n/a	AGCCTGGTCT	ACAGAGTGAG	TCCAGTCTCT	CTCCTTCCCT	3	TCT	
Sim47	n/a	TTGGAGGTCT	GAAGAGCGCT	GGTGTGGGCC	ACCACATCCA	0	No_MH	
Sim48	n/a	AGAAAAAGTT	CAAACTAGAA	TTATAGGAAT	GAGCAACAAT	1	T	
Sim49	n/a	AGTGTCATGT	GACGTCAAGG	TATCTTCCAG	GTTCAAAACA	1	G	
Sim50	n/a	GTACAGATTG	TACATTGTTC	ATATTTTAAG	GTCATGTTGT	1	G	
Sim51	n/a	AAAACAAATG	TATAGATAAA	TGCAGACACA	GGAGCTATCT	0	No_MH	
Sim52	n/a	CACCTCTCAG	ATATCTCCTG	TATGGAGAGC	TGTGGCCCTG	0	No_MH	
Sim53	n/a	AGGAAAAAGA	AACAGTGATA	TTCCTCAATG	GCTTCTTTCC	0	No_MH	

Sim54	n/a	CAGTGTTTTT	TGTTTTTTTG	ACCTTAAGGG	AGAGGGGAAA	0	No_MH
Sim55	n/a	CTTCATCACT	CCCAGGCTTA	TAAAAACCTT	TTCACCAACT	1	T
Sim56	n/a	GCCCTAAGTA	AAGAAGTTGC	CTTAATTCTT	TTCAGACTCT	0	No_MH
Sim57	n/a	GTACAATTTC	CTTTTTCCTC	AGATAGGACA	GTGTTCAAAC	0	No_MH
Sim58	n/a	CCATCTGTGG	CCAGCCAGGC	TAGCCTAAGT	TTTACAGTCT	0	No_MH
Sim59	n/a	CTTCCCCTTC	TGTTTTCCCA	TATCTGGTCG	CTTAGTGCTA	0	No_MH
Sim60	n/a	TTGCAAAGGG	AACAAGGATG	CCAAGGATCA	TAGAAGCGAG	0	No_MH
Sim61	n/a	CTAAACTGCA	ACACCGGATA	CTAACATGTT	GCTTGGTAGG	0	No_MH
Sim62	n/a	CTCAGCACCT	GACTCAGGGC	AAAAATGGTG	CTTTCTTTGC	0	No_MH
Sim63	n/a	CAAATTCTCA	AACATATTGA	AAAGTATGAA	ATTGTAGAAA	1	A
Sim64	n/a	AAGTGGATTC	TCACATTCAT	AGAACAGTTT	ATTCCAGGGA	0	No_MH
Sim65	n/a	TTTTGTTAAA	GATATTTACT	TGAGAATATC	*ATCCTGAGTG*	0	No_MH
Sim66	n/a	AGAGATCTGA	CAGGGTTGGG	CGGTTTTTTG	TTTGTTTGTA	0	No_MH
Sim67	n/a	TGTGTTTGTT	CACACACATG	GCATACTCTG	AAGAGCTCCG	0	No_MH
Sim68	n/a	TGATCTGATG	TAAGCCTCTG	TGGAACAAAA	CATAGCAACC	0	No_MH
Sim69	n/a	CTACCTCTGC	CTCCCAGGCT	CAGCCCCCTT	GTTTTAGAAT	0	No_MH
Sim70	n/a	GATCTCATTA	CGGATGGTTG	GCTGTCCTGG	AACTCACTTT	0	No_MH
Sim71	n/a	CCTCAACCCA	AAATAAAATG	GGCACCATCT	CCACAGCAGT	0	No_MH
Sim72	n/a	GATCTTGCAG	AATAACTTAC	AGAACAAATA	ACAGGGCTTT	1	A
Sim73	n/a	TTAGGTTCTG	GCAGAGTCTT	TGTATTTTTC	TTAGTTTTCT	0	No_MH
Sim74	n/a	GCAAGTACAT	ATCCCTCACA	AGGGTAGGGG	GAGGGTATAG	0	No_MH

Larger deletion sequences from literature

ID	Site	Upstream (retained)	Upstream (lost)	Downstream (lost)	Downstream (retained)	MH score	MH	Notes
1	*Ngn3*	CTCCGAGGAG	GTACTCGACC	CAAAGGGCAG	GGGTCGGGGG	2	AG	Adikusuma'18
2	*Ngn3*	CCCGACCCCT	GCCCTTTGTC	GGCAGGGCAC	GCTCCTGGCC	2	GC	Adikusuma'18
3	*Ngn3*	CGACCCCTGC	CCTTTGTCCG	CACGACTTGC	ATGCAGCTCA	3	TGC	Adikusuma'18
4	*Ngn3*	CTGGATTCCG	GACAAAGGGC	CAGCTCTCCG	CCACTATGGG	4	TCCG	Adikusuma'18
5	*Ngn3*	AGCTGGATTC	CGGACAAAGG	CACCATAGTC	CTCATCCGAG	2	TC	Adikusuma'18
6	*Ngn3*	AGGTCAGTCA	GTGACAAGCA	GCAGCACTCT	GTTTGTTCCC	2	GT	Adikusuma'18
7	*Ngn3*	CTCCCGACTC	AAAAGAACAG	GACAAAGGGC	AGGGGGGAGC	1	A	Adikusuma'18
8	*Ngn3*	TTCGGAGCAG	TGAGAGTGGG	CAAAGGGCAG	GGGTCGGGGG	4	GCAG	Adikusuma'18
1	*Foxp4*	AAGGGGTGAC	CCGGCTTAGC	CAGGGGTGAC	AGGTCAGGGA	9	AGGGGTGAC	Adikusuma'18
2	*Foxp4*	TCCCATTGTC	CTTGGGTTGC	TCCAACTGGA	GATGGAGCCC	0	No_MH	Adikusuma'18
3	*Foxp4*	AGCCCCAGCG	TTCCCATTGT	GGGACTCCCC	TTTCCATGGC	2	TT	Adikusuma'18
4	*Foxp4*	CCTGCAACCC	AAGGACAATG	TTTAGGCAAA	ATAAGGCTCC	1	A	Adikusuma'18
5	*Foxp4*	CACAGGTGCC	CTTTGGGTTT	TCCCATTGTC	CTTGGGTTGC	3	CTT	Adikusuma'18
6	*Foxp4*	GCTGTGAGAG	GTGTGCCCCT	TTGTCCTTGG	GTTGCAGGGG	2	GT	Adikusuma'18
7	*Foxp4*	CCCCTGCAAC	CCAAGGACAA	GACATTTAAC	GACAGGGTGA	3	AAC	Adikusuma'18
1	*Fzd3*	ACACTAGCAC	GTGCACCAAT	AAAAGGGGAC	AGTGTCAAAG	2	AC	Adikusuma'18
2	*Fzd3*	AGCTTAGCTT	AAGGGTGTGA	CAAACTTTTA	AGAATTGTTT	1	A	Adikusuma'18
3	*Fzd3*	GGATAGAAAA	TTCTATACTT	AAGGGGACAG	TGTCAAAGGC	1	T	Adikusuma'18
4	*Fzd3*	CTCTATCTAT	CTATAACATA	CAAAATCTGA	CTCAGGAGAA	2	CT	Adikusuma'18
5	*Fzd3*	CCAAGCTTAG	CAAGGGTGTG	TATTCACTTT	CATACGTCAA	2	CA	Adikusuma'18
6	*Fzd3*	TAGCAAGGGT	GTGAAAAGGG	TTTCTGGAAC	AATAAAGCTT	0	No_MH	Adikusuma'18
7	*Fzd3*	TACTATATAT	GGTATAACCC	GTGTGAAAAG	GGGACAGTGT	2	GG	Adikusuma'18
8	*Fzd3*	GCTTAGCAAG	GGTGTGAAAA	ACTCACTAAG	TGTGAACCAA	3	AAG	Adikusuma'18
9	*Fzd3*	TCCAAGCTTA	GCAAGGGTGT	AAAATCTAAA	ACTCCTATTC	1	A	Adikusuma'18
10	*Fzd3*	CCGAGTATAG	GAAGTTTACA	GTGTGAAAAG	GGGACAGTGT	2	AG	Adikusuma'18
1	*Viperin*	CCCCGGACCT	GTGGCTGTTC	GAAAATGAAA	GTTGGACCAG	2	GT	Adikusuma'18
2	*Viperin*	TTAAAAATTG	TTGAGTGTGC	GAAGGAACAG	CCACAGGTCC	1	G	Adikusuma'18
3	*Viperin*	GGGTGGCTAG	ATCCCGGGAA	TCCAGGACAG	TTGTGCATAG	2	AG	Adikusuma'18
4	*Viperin*	TGGGTGTCCT	CCGGCTCACC	TGCTCTTTCC	CCGCCCACCC	3	CCG	Adikusuma'18
5	*Viperin*	GGACCTGTGG	CTGTTCCTTC	AGCAGCCGAG	CAGCTAGAGC	1	C	Adikusuma'18
6	*Viperin*	CACCCCGGAC	CTGTGGCTGT	AAAGTTGGAC	CAGAGCCAAG	4	GGAC	Adikusuma'18
7	*Viperin*	GGGGCGTGGC	TAGCTGGGCC	TGGAACTCAC	TCTTGTTGGC	1	T	Adikusuma'18
8	*Viperin*	CATAGACTTG	CTGTACCCAG	CGGGATCTAG	CCACCCTAAT	1	C	Adikusuma'18
9	*Viperin*	CAGGTGTGTG	CCTATCACCA	CTATTCATCC	TTGTGTTTGG	0	No_MH	Adikusuma'18
10	*Viperin*	GGACCTGTGG	CTGTTCCTTC	AGCAGCTAGA	GCAGTGGGCA	0	No_MH	Adikusuma'18
1	*Pik3r6*	GTGGAAGTGG	GGAAGGTAAC	ATTGCTCTGG	AGGGCTGGGT	3	TGG	Adikusuma'18
2	*Pik3r6*	TTTGGAAGTG	GAAGTGGGGA	GATTGCTCTG	GAGGGCTGGG	2	GA	Adikusuma'18
3	*Pik3r6*	ACGGGATAGG	GGCTGGGGGA	AATCCATCAG	GAGTTGGCAA	1	G	Adikusuma'18
4	*Pik3r6*	CCCTGATTGC	TCTGGAGGGC	GGGCATATGC	ACATGTATGT	3	TGC	Adikusuma'18

1	*Hmgcs2*	TACTGTGCAG	AATAAATTAA	GCCAGGGGAG	CAGGAGGGAT	2	AG	Adikusuma'18
2	*Hmgcs2*	TAAGAACCAG	TACTTCTGTG	TGTCTTTTGA	CCAGTTACCT	0	No_MH	Adikusuma'18
1	*ApoE*	CCAACCCTGG	GAGCAGGCCC	GCCAGGTTGG	CCTTGAACTC	3	TGG	Ma 2014
2	*ApoE*	TTGATGCTCA	GAGGGTAAAG	CTGGGAGCAG	GCCCTGAACC	1	G	Ma 2014
3	*ApoE*	CACTGTAGGT	CCTGACCCAG	GCCCTGAACC	GCTTCTGGGA	0	No_MH	Ma 2014
4	*ApoE*	CCTTAAACTT	ACTACTCTAC	TGAACCGCTT	CTGGGATTAC	3	CTT	Ma 2014
5	*ApoE*	TGACCCAGCC	TTAAACTTAC	CTGAACCGCT	TCTGGGATTA	1	T	Ma 2014
6	*ApoE*	CCCTGGGAGC	AGGCCCTGAA	AGTGCTCAGC	GCTTCACCCT	3	AGC	Ma 2014
1	*Prf1*	CACTGCCAGC	GTAATGTGGC	CCAAGATAGC	CAATTTTGCA	3	AGC	Ma 2014
1	*B2m*	GGAACAGAGG	AACAGTTGTA	CAAGGACTGG	TCTTTCTACA	2	GG	Ma 2014
2	*B2m*	GTAGGAAAAA	CAGAAGGTGT	CTGTCCTTCA	GCAAGGACTG	1	A	Ma 2014
3	*B2m*	AACTCCCCAA	ATTCAAGTGT	CCGATCCGTA	GTGCCAGCAG	1	A	Ma 2014
4	*B2m*	AAACTCCCCA	AATTCAAGTG	CCCAAAACCG	TCACCTGGGG	0	No_MH	Ma 2014
1	*Prkdc*	CTGTTCAGAA	ACACCAAGGA	AGTACTGAGA	TTACAAATGT	1	A	Ma 2014
2	*Prkdc*	TGTGATTCAT	GGTGGGCCCC	TTCTTGTTCT	TTTTGATACA	1	T	Ma 2014
3	*Prkdc*	TCAGGTGAGT	ACTATTTTAA	TGATTCATGG	TGGGCCCCAG	0	No_MH	Ma 2014
1	*B2m*	TTCTAGTTCA	TGAAACCAGA	CGCCTCACAT	TGAAATCCAA	5	TGAAA	Zhou 2014
1	*Prkdc*	TGTGATTCAT	GGTGGGCCCC	CTGAATTCAT	TTAAAGCACA	6	ATTCAT	Zhou 2014
2	*Prkdc*	TACGTTCTCT	ACTAAAGTTT	ACCAAGGACT	GAAGCTTGCA	2	CT	Zhou 2014
1	*Kcnk13*	CAACCCTCGC	GGAGCTGTCC	CAACCTGAGC	CGTGAAGAGC	2	GC	Mianne 2017
2	*Kcnk13*	TGGCTGGCCG	CGGTTGCGGC	TGCGCCCTCG	CTGGGACTTC	2	CG	Mianne 2017
1	*Tyr*	AGTGCTCAGG	CAACTTCATG	ATATCTCAGG	GCAGAAAATG	6	CTCAGG	Parikh 2015
2	*Tyr*	GACCTCAGTT	CCCCTTCAAA	CACCAGGGTT	TCTGCCTTGG	3	GTT	Parikh 2015
3	*Tyr*	TCAATTCAAG	ATAATCAAGG	AGGCAACTTC	ATGGGTTTCA	2	AT	Parikh 2015
4	*Tyr*	CTCAGTTCCC	CTTCAAAGGG	TCATCCCCAC	AGGCACCTAT	1	C	Parikh 2015
5	*Tyr*	CTCAGTTCCC	CTTCAAAGGG	GAAAATGTGC	CTTACTAACA	3	CTT	Parikh 2015
6	*Tyr*	AACAGGCTGA	GAGTATTTGA	CATAGAATCA	TTGACTTTGT	1	A	Parikh 2015
1	*Dip2a*	CCCCTCAGCC	GCGCACGCGC	TGAGCCACCG	GCCATGGCCG	2	GC	Zhang 2015
1	*Tet1*	AACTAATCCA	AACAAAGAGA	AGGGAGCTCA	TGGAGACTAG	2	CA	Wang 2013
1	*Tet2*	GTGCCAACAG	ATATCCAGGC	TGCAATCCAG	GTACATGCCA	3	CAG	Wang 2013

Simulated larger deletion sequences (Supplementary Figure 5)

ID	Site	Upstream (retained)	Upstream (lost)	Downstream (lost)	Downstream (retained)	MH score	MH	Notes
Sim1	n/a	ATATGAAGGG	ATAGCCGGTC	TAGCCTAAGT	TTTACAGTCT	0	No_MH	
Sim2	n/a	ATAGTGAATT	TCCTAAATTA	TATCTGGTCG	CTTAGTGCTA	0	No_MH	
Sim3	n/a	CATGGTGCTG	GAGAAGTAGC	GGCTACACAG	AGAAACCCTG	1	G	
Sim4	n/a	CTTGCTTCAT	TCTTGTGACG	GTGTCTCCTG	TCCCTCCTGT	2	TC	
Sim5	n/a	GGGATCTTTG	ACATGCCTGC	TTGGGTACTT	GTTCAATCAC	0	No_MH	
Sim6	n/a	CCAGAATTAA	TCATTTTGTG	TCCTTTTTGT	TTGATTCTCT	1	T	
Sim7	n/a	AAGATGGAAG	GAATATAGTG	GAGCACTGCA	TTTGTCATTG	0	No_MH	
Sim8	n/a	CAGAGGAAGT	AAGCGAGATA	TGTTCCCATC	CCTGACTTGG	0	No_MH	
Sim9	n/a	TCTCACATAA	GACTCTAAGC	GGGACAGAGA	GCCTAAGGGA	1	G	
Sim10	n/a	ACAGCTGAGC	TAAAGCAGAC	TATTTCTCCT	CCCAGCCCAA	0	No_MH	
Sim11	n/a	GCCATGCCCT	TGAAAGCCAA	TCCTATCCCT	GCCTCAGCTG	4	CCCT	
Sim12	n/a	ATTTCTAGAT	TTTCATATTC	CAAGGACTTG	TAATGTAAGT	1	T	
Sim13	n/a	TGCCTCCTAT	TCTGGCTTTT	GGTGAGAAAT	GGGCAGGGAG	2	AT	
Sim14	n/a	CCCTGTGATG	CAGTGACCAT	AAAAACCCCA	CTAGATCTAA	1	C	
Sim15	n/a	AAATTGGTCT	GAAGTTCTCT	TATGGTCAAT	TTTGGAGAAG	1	T	
Sim16	n/a	ATTGTACCTC	ACACAATAAT	AGTCTCCCAG	CCAAAAAAAA	0	No_MH	
Sim17	n/a	AGTCCAGTAA	CTCCTGCATC	TCTCCGTCAC	ACTGTTACCA	0	No_MH	
Sim18	n/a	ACACCCGGCT	CAAAACTTTT	CTCAGTTTAG	ACAGGTTGCT	0	No_MH	
Sim19	n/a	TTGTTGAAAA	TGCTGTCTTT	TTGTCAAATG	CTTTCTGTGC	0	No_MH	
Sim20	n/a	TTCCTGGTTT	CCCTCCGAAA	CGGGGTGAGA	TTAAGAATGT	0	No_MH	
Sim21	n/a	TCTCGAGGAA	AGATGGGGCC	CTGCTCTGAC	ATGCATACAG	1	A	
Sim22	n/a	GCCTACTTGT	GTTTACTTCT	AATAGATATC	TCAGAAAGAG	0	No_MH	
Sim23	n/a	ACCTCAGCTA	GTGACAGTTG	GAGGTAAGCC	AGGGCTGGGC	0	No_MH	
Sim24	n/a	CCCTGGCCCT	AATCCTATGC	AGAAATCAAC	CTTGATATTG	0	No_MH	
Sim25	n/a	GCTCATCCTC	TAACACACAA	AACCAGTAGT	CAGAGCTGGA	0	No_MH	
Sim26	n/a	TCCAGTTTCT	TTGAGCTCTA	GAAGGAATTT	TAATCTTAGG	1	T	
Sim27	n/a	CATCGTAAGA	TCTCTTTGTA	AGGAGATGGT	AACCTCATAC	0	No_MH	
Sim28	n/a	TTTTTTTTCA	CTACAAAATG	AGGGAGAGAG	AATTTCCTAG	0	No_MH	
Sim29	n/a	TCAGGCCTTA	AATACATTAT	CAAGTTGATA	TAGTACTTAT	2	TA	
Sim30	n/a	CATGTGCCCA	TGGGCGCACA	AAGTGGTGGC	AGGCAGCATC	0	No_MH	

Sim31	n/a	GTATTTCTGT	AGACGTTTTA	CTATAAATTA	TATAGTACTG	0	No_MH
Sim32	n/a	TCCAAGTGCC	TAGAACTACA	TGGGATTTTT	TTCTCCTTCA	1	T
Sim33	n/a	GTGGATCTCT	GTAAGTTTGA	TTTTGTTAAG	TTACACTATA	0	No_MH
Sim34	n/a	CTCTCCAAAG	CTCTGAAGGA	TCCAAGAGGC	AGGGGCAGGT	0	No_MH
Sim35	n/a	AGCCCCCATC	TTACAGATGG	CTGAGCCATT	TGCCAGCTAT	1	T
Sim36	n/a	GAGTTGGTGC	TCATTTTCCT	TCTTTACTCT	AACCCAGAGA	0	No_MH
Sim37	n/a	GTGGATTATG	AAAATAGAAA	TGTGGAGTCT	GAACTGGCGA	0	No_MH
Sim38	n/a	CCACCGGACA	AAAGGCAGAA	GCGTAGCTAT	ACCCACAGCA	1	A
Sim39	n/a	CATCTTTTCC	CCATCCATTC	CATCCACAGT	AAAAGCCAAA	0	No_MH
Sim40	n/a	CCATCCTTTC	AAAAAAAAAA	GTGTATGCTC	TTAACACTAG	2	TC
Sim41	n/a	ACCCATTAGA	CCCAGAATGC	AGATTGATGA	CTCAGCAGTT	2	GA
Sim42	n/a	ATTATCTTCC	TCTCGCCCTT	CAGAGAGCTA	CCTGTCTCTT	0	No_MH
Sim43	n/a	TTATTTTCTT	CATTCTTTGT	GGTGCCATTC	TCTTCTGGAA	0	No_MH
Sim44	n/a	ATAATCCAGC	AACATGAGTG	GGTGTTGAGA	AGGACTGGTA	1	A
Sim45	n/a	CCAGCTAGCT	TCCCTGCTGA	TTCAGTTCAA	GTTAGTTTCA	0	No_MH
Sim46	n/a	GGAGATGGAA	TTTTTGCCAA	GTGTTAGGAG	TGGATGGGGA	1	T
Sim47	n/a	GGATCCCATG	AGAGGCTTCA	CTGGAATTCA	AGCAGTTGCA	2	AG
Sim48	n/a	GAAGGGTATG	AAAGAACCTG	CTGCTGGGTG	GCCAAAGTTA	2	TG
Sim49	n/a	CAGGGCATGT	CCTCCATCAC	GCCGTCCTGT	TCTTAGCCAC	3	TGT
Sim50	n/a	GTTAAAGATT	TATTTATTTA	TGAAAAAGCA	TTATTTCAAA	1	T
Sim51	n/a	GATTGGTTCC	TAACATCCAC	TGGTCCTCTG	GAGTCTAACT	0	No_MH
Sim52	n/a	ATCTCGCTCC	AGAATAACCT	AGCAGCTATG	GAATTAGCCG	0	No_MH
Sim53	n/a	AAATGGAAGA	GAGAATTTCA	TTCCAACCCA	AAGTTATCAA	1	A
Sim54	n/a	AAGAAAATAC	AAAGGAGGCA	CTTAAGGCTT	TGTTCCTACA	0	No_MH
Sim55	n/a	CTTTGGGGTC	CTTGAGCCAG	GTATCCTGGG	AAGTCCATGC	0	No_MH
Sim56	n/a	TACATGGGCA	CAGGGGAAAA	TTAAGGGAAG	TAGAGGGTGG	0	No_MH
Sim57	n/a	AACTTAAGGG	ACGTGGAAAA	ACCTTGAACT	TGTGTTCCTG	0	No_MH
Sim58	n/a	ATGGAATACC	ACTCAGCTAT	AGACAGGGCA	TCAAGTGGAT	0	No_MH
Sim59	n/a	GCTTTTAGGT	ATATTTTTGT	GCTCCAGCTG	GATATGTAGC	0	No_MH
Sim60	n/a	TTAAATCATC	ACCCTACCCT	GGTTTCTCTG	TATAGCCCTG	0	No_MH
Sim61	n/a	TTCATTTCCA	GAGCACTTTG	AGACATTTCC	TTTTGTTCCC	0	No_MH
Sim62	n/a	TACAGCACTC	CCATCCCCGC	TGCCGGGAAG	GGGAATTGCT	0	No_MH
Sim63	n/a	GATACACAAA	GCTTCATCTG	ACCTGCCCTG	CGCACATACT	0	No_MH
Sim64	n/a	ATATCAAAGC	CACTGACTTA	CAAGGCCCCA	GGACACAAGA	0	No_MH
Sim65	n/a	TTAACAGTAC	TACTGATTCC	AACAGCATGC	ACAAGACCTG	1	C
Sim66	n/a	CTCCAGCCCC	CTGGTGACTT	AGGCAGCAGG	ATCAGGAGTT	0	No_MH
Sim67	n/a	CCCCTCACTG	GGGGATTCTA	TCAGAGTGAG	ACACCCTGTC	1	G
Sim68	n/a	TGCCCATTGA	CACTACCTGG	TAAGTTGCTG	AGTGGGTCCT	0	No_MH
Sim69	n/a	CCAGCCCTGA	AGGAATTTTT	AACTAGGAAG	AGGTTAAGTT	3	AGG
